# Bioprospecting of UVB/UVA Absorbers in Fungi Associated with the Macroalga *Phaeurus antarcticus* Led to the Isolation of Isocoumarins and a Benzofuran with Photoprotective Potential

**DOI:** 10.1021/acsomega.6c03690

**Published:** 2026-07-02

**Authors:** Gustavo Souza dos Santos, Karen Cristina Rangel, Izadora de Souza, Ana Júlia Pasuch Gluzezak, Maria Valdeline Sousa Teixeira, Ana Carolina Jordão, Isadora de Jesus da Silva, Ludmilla Tonani, Niege Araçari Jacometti Cardoso Furtado, Pio Colepicolo, Marcia Regina von Zeska Kress, RuAngelie Edrada-Ebel, Lorena Rigo Gaspar, Hosana Maria Debonsi

**Affiliations:** † Department of Biomolecular Sciences, Faculty of Pharmaceutical Sciences of Ribeirão Preto, University of São Paulo, Prof. Dr. Zeferino Vaz Avenue, Ribeirão Preto, SP 14040-903, Brazil; ‡ Department of Pharmaceutical Sciences, Faculty of Pharmaceutical Sciences of Ribeirão Preto, University of São Paulo, Prof. Dr. Zeferino Vaz Avenue, Ribeirão Preto, SP 14040-903, Brazil; § Department of Biochemistry, Institute of Chemistry, University of São Paulo, Prof. Lineu Prestes Avenue -748, São Paulo, SP 05508-000, Brazil; ∥ Department of Clinical Analyses, Toxicology and Food Science, Faculty of Pharmaceutical Sciences of Ribeirão Preto, University of São Paulo, Prof. Dr. Zeferino Vaz Avenue, Ribeirão Preto, SP 14040-903, Brazil; ⊥ Strathclyde Institute of Pharmacy and Biomedical Sciences, University of Strathclyde, 161 Cathedral Street, Glasgow G4 0RE, U.K.

## Abstract

Antarctic organisms are exposed to intense ultraviolet radiation (UVR) during austral summer, and although their photobiology remains poorly explored, their secondary metabolites play a key role in UV resistance, representing a promising source of novel photoprotective compounds. In this study, endophytic fungi associated with the seaweed *Phaeurus antarcticus* were screened for their ability to produce photoprotective metabolites. Four fungi were isolated and identified by ITS sequencing as *Penicillium* sp. (LMC 8102), *Cladosporium* sp. (LMC 8108), *Rhinocladiella similis* (LMC 8103), and *Epicoccum* sp. (LMC 8106). Among them, *Penicillium* sp. showed the highest UV absorbance and photostability. To enhance metabolite production, a culture media optimization was applied. Bioguided fractionation of the optimized extract led to the isolation of four compounds, 5,6,8-trihydroxy-4-(1-hydroxyethyl)-1H-isochromen-1-one (**1**), aspergillumarin A (**2**), sescandelin (**3**), and vermistatin (**4**). All compounds exhibited absorption bands within the UVB (280–320 nm) and UVA-II (320–340 nm) regions, particularly between 280–300 nm and 320–340 nm and were photostable. Compounds **2**–**4** maintained HaCaT cell viability above 79% under UVB exposure, while sescandelin (**3**) reduced UVA-induced ROS generation by 15% in HaCaT cells and 36.5% in reconstructed human skin. Photosafety evaluation using photoreactivity (OECD TG 495), phototoxicity (3T3 NRU PT, OECD TG 432), and ocular irritation (HET-CAM) assays demonstrated that compound **3** was nonphototoxic, whereas none of the evaluated compounds (**2**–**4**) exhibited photoreactivity. Compounds **3** and **4** did not induce ocular irritation in the HET-CAM assay. Overall, sescandelin (**3**) demonstrated the most promising photoprotective, antioxidant, and safety profile, supporting its potential application in topical sunscreen formulations.

## Graphical abstract

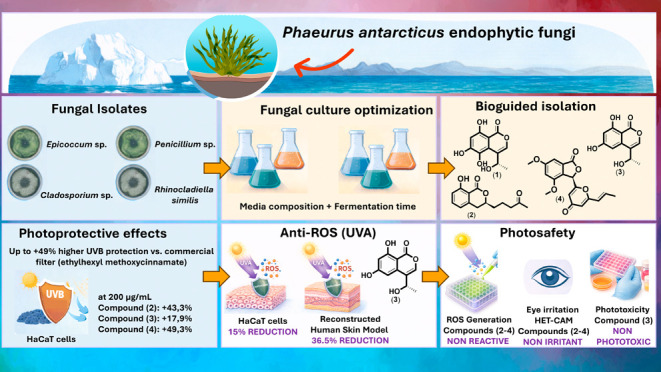

## Introduction

Sunlight exposure stimulates the production of vitamin D and β-endorphins in human skin, conferring several health benefits.^1^ However, excessive exposure to ultraviolet radiation (UVR) is associated with several adverse effects, including premature skin aging, immunosuppression, oxidative stress, and skin carcinogenesis.^2^ Based on wavelength, UVR is classified into UVC (200–280 nm), UVB (280–320 nm), and UVA (320–400 nm). Exposure to these wavelengths may induce DNA lesions directly or trigger oxidative stress through the generation of reactive oxygen species (ROS), resulting in a range of molecular and cellular alterations.^3^ While the ozone layer efficiently absorbs UVC radiation, approximately 5.1% of solar UVR reaches the Earth’s surface, consisting predominantly of UVA radiation and smaller amounts of UVB.^4^ Prolonged UVA exposure is associated with hyperpigmentation, photoimmunosuppression, inflammation, and actinic keratosis, whereas UVB is strongly associated with erythema induction, photoaging, and skin carcinogenesis.^5,6^

To mitigate UVR-induced skin damage, sunscreens constitute one of the primary photoprotective strategies. However, some synthetic UV filters have raised concerns regarding potential toxicological and environmental impacts, including endocrine-related effects, placental transfer, and possible ecotoxicological effects on marine ecosystems.^2,6–8^ Although current evidence supports the safety of approved UV filters within established regulatory limits,^6^ there is growing interest in identifying novel photoprotective compounds with improved safety, stability, and multifunctional properties, particularly those inspired by natural products.

Marine organisms are continuously exposed to solar radiation and have evolved diverse protective mechanisms against UV-induced damage. In polar regions such as Antarctica, seasonal ozone depletion and elevated UV exposure represent additional environmental stressors that may influence the biosynthesis of adaptive secondary metabolites.^3,9^ In addition to UV exposure, marine microorganisms are subjected to salinity fluctuations, osmotic stress, and low temperatures, conditions that may contribute to the production of chemically diverse metabolites with ecological and protective functions. Consequently, marine-derived microorganisms, particularly fungi associated with macroalgae, have emerged as promising sources of structurally unique bioactive compounds.

Marine-derived compounds have attracted attention as potential photoprotective agents due to their UV-absorbing chromophores, antioxidant properties, and photostability.^10^ Metabolites such as alkaloids, isocoumarins, and mycosporin-like amino acids have been highlighted as promising candidates for sunscreen-related applications because their conjugated aromatic systems and chromophoric structures enable efficient UV absorption and dissipation of excess energy.^10–12^ In marine and Antarctic environments, these metabolites may play adaptive ecological roles related to photoprotection and oxidative stress defense.

In addition, marine-derived fungi represent attractive targets for natural product discovery because they can be cultivated under controlled laboratory conditions, enabling scalable production and facilitating the investigation of environmentally induced metabolic responses, unlike many marine macroorganisms such as algae and sponges, which are often difficult to cultivate and maintain under laboratory conditions.^13^

Marine-derived fungi are increasingly recognized as a promising source of secondary metabolites with potential photoprotective properties. Previous studies have described UV-absorbing metabolites exhibiting photostability, antioxidant activity, and in vitro safety from the Antarctic algal-associated fungus *Arthrinium* sp., isolated from the macroalga *Phaeurus antarcticus*.^14^ Similarly, *Penicillium echinulatum*, an endophytic fungus associated with the Antarctic macroalga *Adenocystis utricularis*, was shown to produce quinoline alkaloids such as viridicatin, which exhibited UV absorption, antioxidant activity, and photostability, supporting the potential of marine fungal metabolites for photoprotective applications.^15^

In contrast to conventional commercial UV filters, several marine-derived metabolites may combine UV absorption with antioxidant and photostability-related properties, supporting their investigation as multifunctional photoprotective candidates.^16^

This study aimed to investigate endophytic fungi associated with the Antarctic seaweed *P. antarcticus* as a source of UV-absorbing metabolites with potential photoprotective applications. By evaluating their antioxidant activity and photosafety using nonanimal approaches, this work highlights marine-derived fungal metabolites as promising candidates for future photoprotective formulations and skin-care applications.

## Experimental Section

### Algae Collection and Fungal Isolation

Healthy specimens of *P. antarcticus* were hand-collected during low tide at three sites in the South Shetland Archipelago, Antarctica: King’s George Island (62°07.560′ S, 58°23.626′ W), Half Moon Island (62°35.383′ S, 59°54.957′ W), and Greenwich Island (62°26.652′ S, 59°43.545′ W). Sampling was conducted during the Brazilian Antarctic Expedition OPERANTAR XXXIV in 2015. Expedition logistics were supported by the Brazilian Antarctic Survey (PROANTAR) and the Brazilian Navy.

The samples were identified based on macro- and micromorphological analyses. A voucher specimen was deposited in the Maria Eneyda Kauffmann Fidalgo Herbarium, Botanical Institute of São Paulo, São Paulo, Brazil, under voucher number SP 470404. After collection, *P. antarcticus* samples were rinsed with sterile seawater to remove adherent debris. To eliminate epiphytic microorganisms and allow the isolation of fungi associated with the inner tissues of the seaweed, the samples were surface disinfected prior to the isolation procedures.

Initially, the seaweed samples were cut into small fragments (0.5–1.5 cm) and surface-disinfected using three different protocols, designated methods I, II and III (MI – MIII), as previously reported and established at the Laboratory of Organic Chemistry of the Marine Environment.^17^ In Method I (MI), algal fragments were immersed in 70% (v/v) ethanol for 15 s. In Method II (MII), fragments were sequentially treated with 70% (v/v) ethanol for 5 s and 2.5% (v/v) sodium hypochlorite for 5 s. Method III (MIII) consisted of sequential immersion in 70% (v/v) ethanol for 10 s followed by 2.5% (v/v) sodium hypochlorite for 10 s. After each treatment, the fragments were rinsed three times with sterile seawater.

To verify the effectiveness of the surface-disinfection procedures, treated fragments were first gently pressed onto the surface of the isolation medium to obtain imprint controls (Control 1) and subsequently transferred to fresh Petri dishes containing potato dextrose agar (39 g/L) prepared with sterile seawater and supplemented with chloramphenicol (200 mg/L). Cultures were monitored for up to 60 days. Control 2 consisted of plating aliquots from the final rinsewater. Fungal colonies that emerged from the algal tissues were purified by repeated streaking and stored at −80 °C until further use.

### Fungal Identification

Fungal biomass was obtained by inoculating material from pure colonies into potato dextrose broth and incubating the culture for 3 days at 23 °C. Molecular identification was performed by sequencing the internal transcribed spacer (ITS) region of the fungal rDNA. PCR amplification were carried out using Phusion High-Fidelity DNA Polymerase (New England BioLabs, Inc.) and primers ITS1 (5′-TCCGTAGGTGAACCTGCGG-3′) and ITS4 (5′-TCCTCCGCTTATTGATATGC-3′).^18^ PCR products were purified using the Wizard SV Gel and PCR Clean-Up System (Promega) and sequenced using the same primers on an ABI3730 DNA Analyzer (Applied Biosystems). Each sequence was analyzed using ChromasPro Software (version 1.7.6, Technelysium Pty Ltd., Tewantin, QLD, Australia). The resulting sequences were then compared with publicly available DNA sequences deposited in the National Center for Biotechnology Information (NCBI) database.^19^

### Screening for UVB/UVA Absorbers

To select a fungal strain capable of biosynthesizing compounds with photoprotective potential, all isolated fungi were cultivated in potato dextrose broth (24 g/L) prepared with natural seawater, and the resulting crude extracts were screened for UV absorption in the 280–400 nm range and photostability following UVA irradiation.

### Small Scale Fermentation for Screening

Fungi were reactivated in potato dextrose agar prepared in sterile seawater. After 7 days of cultivation, 10 plugs (5 mm diameter) of each strain were transferred to four 500 mL conical flasks containing 150 mL of potato dextrose broth (24g L^–1^) prepared in sterile seawater. Cultures were maintained in static conditions for 28 days, at 26 °C.

### Extraction Procedures

After the incubation period, 150 mL of ethyl acetate (EtOAc) were added to each flask containing the fermented broth. Flasks were placed under the fume hood for 24 h, prior isolation procedures. After the maceration period, fungi mycelial mass was fragmented using a glass rod, followed by 5 min in the ultrasonic bath (75 W). The mycelial mass was then removed from the fermented broth by vacuum filtration and the fermented broth submitted to liquid–liquid extraction with EtOAc. The extraction procedure was repeated three times for each culture flask. After phase separation, the aqueous layer was discarded, and the organic phase was concentrated under reduced pressure using a rotary evaporator, maintaining the temperature below 35 °C.

### Optimization of Photoprotective Metabolites Production

#### Fungi Cultivation

The culture medium was prepared using potato dextrose broth (PDB, 24g L^–1^) in either sterilized natural seawater (NSW) or artificial seawater (ASW) consisting of SWBG-11 medium. The artificial seawater composition was as follows: NaCl (25.0 g L^–1^), MgCl_2_·6H_2_O (2.0 g L^–1^), KCl (0.50 g L^–1^), NaNO_3_ (0.75 g L^–1^), K_2_HPO_4_·3H_2_O (0.02 g L^–1^), MgSO_4_·7H_2_O (3.50 g L^–1^), CaCl_2_·2H_2_O (0.50 g L^–1^), citric acid (0.003 g L^–1^), ferric ammonium citrate (0.003 g L^–1^), Na_2_EDTA (0.0005 g L^–1^), and Na_2_CO_3_ (0.020 g L^–1^). The micronutrient solution consisted of H_3_BO_3_ (2.860 g L^–1^), MnCl_2_·4H_2_O (1.810 g L^–1^), ZnSO_4_·7H_2_O (0.222 g L^–1^), Na_2_MoO_4_·2H_2_O (0.390 g L^–1^), CuSO_4_·5H_2_O (0.079 g L^–1^), and Co­(NO_3_)_2_·6H_2_O (0.0494 g L^–1^). The final pH was adjusted to 7.5. *Penicillium* sp. (LMC 8102) was cultivated in 500 mL Erlenmeyer flasks containing 150 mL of culture medium under static conditions at 26 °C in three biological replicates. To evaluate changes in secondary metabolite production, liquid–liquid extractions were performed after 7, 14, and 21 days of fermentation for both media.

#### ^1^H NMR Analysis

Crude extracts of *Penicillium* sp. (LMC 8102) were prepared by solubilizing each sample in 650 μL of DMSO-*d*_6_ (Sigma-Aldrich) to yield a concentration of 5 mg/mL. The solutions were transferred to 5 mm 7″ NMR tubes and then submitted to proton NMR experiments. The experiments were performed using a Bruker AVIII HD 400 (11.7 T). The NMR data were processed using MestReNova x64 software version 14.1.2 (Mestrelab Research S.L.). First, manual baseline correction or Whittaker Smother, apodization and phase correction were performed to all proton NMR spectra. Then, all spectra were stacked and processed under the Chemometrics plugin. Spectral Binning (0.04 ppm); Standard deviation (10%); Normalization (Sum); Data Scaling (None). After processing, the data was exported to a.csv file to be used in further multivariate statistical analysis.

#### Multivariate Analysis

Partial Least Squares-Discriminant Analysis (PLS-DA) was carried out using the software SIMCA ver. 15.0.2 (Umetrics, Sweden). The variables were scaled using the Pareto algorithm in SIMCA software v. 15.0 (Umetrics, Sweden). Partial least-squares discriminant analysis (PLS-DA) was applied to assess the variance between culture media and fermentation time in crude extracts (predictor variables) and the corresponding chemical shift regions (response variables) derived from ^1^H NMR data. Data were normalized by sum to enhance low-intensity NMR peaks, and features with high variability among replicates were filtered using the interquartile range (IQR). Mean intensity values were used to represent metabolite abundance. No data transformation was applied, and Pareto scaling was used for all multivariate analyses.

### Fractionation, Compound’s Isolation, and Identification

#### Crude Extract Fractionation

After media optimization procedures, *Penicillium* sp. (LMC 8102) was cultivated in 80 conical flasks (500 mL) containing 150 mL of PDB (24g L^–1^) prepared with artificial seawater (ASW), whose composition was based on the SW-BG11 formulation for 14 days in static conditions at temperature of 26 °C. After the incubation period, the fermented broth was extracted as previously described. The obtained crude extract (7.0 g) was solubilized in EtOAc, incorporated in silica gel-60 (40–70 mesh), and submitted to vacuum liquid chromatography (VLC). Fractionation was carried out using 800 mL of nine different mobile phases consisting of *n*-hexane (*n*-Hex), ethyl acetate (EtOAc) and methanol (MeOH). A stepwise polarity gradient was applied to yield nine fractions: FrA (Hex 100%), FrB (Hex: EtOAc 9:1), FrC (Hex: EtOAc 4:1), FrD (Hex: EtOAc 3:2), FrE (Hex: EtOAc 2:3), FrF (Hex: EtOAc 1:4), FrG (EtOAc 100%), FrH (EtOAc: MeOH 7.5:2.5) and FrI (MeOH 100%).

#### Isolation of Compounds 1 and 2

Fraction FrE (350 mg) was submitted to classical column chromatography using a Waters Sep-pak Vac 35 cc Silica 20 g (40–70 mesh). Elution consisted of a three steps gradient (200 mL), *n*-hexane: EtOAc (6:4); EtOAc: *n*-hexane (8:2) and MeOH: EtOAc (2:8). A total of 15 sub fractions were collected. After thin layer chromatography (TLC) analyses, fractions were reunited into 10 subfractions (Fr1 – Fr10). Subfraction FrE-6 was further purified by semi preparative reverse phase High Performance Liquid Chromatography (HPLC; LC-6AD model, Shimadzu) coupled with diode array detector (SPD-M10A model, Shimadzu) with a semipreparative column C-8 (25 cm x 10 mm, 10 μm—Ascentis Supelco, USA). The mobile phase consisted of a polarity gradient method changing the concentration of acetonitrile (ACN) in water: 40% (0 min) – 50% (7 min) – 100% (10 – 12 min) – 40% (16 min) at a flow rate of 3.5 mL per minute injection volume of 500 μL and monitoring at 254, and 329 nm.

#### Isolation of Compounds 3 and 4

Fraction FrF (600 mg) was submitted to flash chromatography carried out in the Isolera Prime (Biotage) coupled to a UV detector. Silica Cartridge (25 g, 40–70 mesh) was used as a stationary phase. The elution was carried out in gradient mode using a mixture of *n*-Hex and EtOAc as follows: 20% EtOAc during 5 column volumes; 20% to 80% EtOAc in 10 column volumes; 80% EtOAc during 5 column volumes. The flow rate was set to 17 mL/min and automatic collection of 14 mL. A total of 54 subfractions were collected and then analyzed by TLC. After comparison, subfractions were pooled to yield a total of 14 subfractions (FrF-1 – FrF-14). Subfraction Fr4 purification was carried out by semi preparative reverse phase HPLC (LC-6AD model, Shimadzu) coupled with diode array detector (SPD-M10A model, Shimadzu) with a semipreparative column C-8 (25 cm x 10 mm, 10 μm—Ascentis Supelco, USA). The mobile phase consisted of a polarity gradient method changing the concentration of acetonitrile (ACN) in water: 35% (0–5 min) – 100% (20 min) – 35% (25 min) at a flow rate of 3.5 mL per minute, injection volume of 500 μL and monitoring at 254, 270, and 340 nm.

#### Structure Identification

Structural elucidation of the isolated compounds was achieved by combining high-resolution mass spectrometry (HRMS) and one- and two-dimensional nuclear magnetic resonance (NMR) analyses. HRMS measurements were performed using a Bruker microTOF-Q II mass spectrometer equipped with an electrospray ionization (ESI) source and time-of-flight (TOF) analyzer. NMR experiments, including ^1^H, HSQC, HMBC, and ^1^H–^1^H COSY, were carried out at the Department of Chemistry of the Faculty of Philosophy, Sciences and Letters at Ribeirão Preto (USP) on a Bruker DRX-500 spectrometer operating at 500 MHz. Spectra were recorded in deuterated methanol (CD_3_OD), dimethyl sulfoxide (DMSO-*d*_6_), or chloroform (CDCl_3_) purchased from Sigma-Aldrich. Compound identification was established through interpretation of the spectroscopic data and comparison with values previously reported in the literature.

### Photoprotective Potential Assessment

#### Measurement of the UV Absorption Spectra

The UV absorption spectra (280 – 400 nm range) of the crude extracts, fractions and pure compounds in methanol (100 μg/mL) was obtained by using a spectrophotometer (Agilent 8453). This analysis was performed in triplicate and the experimental data was plotted using the software Origin 8 (Origin Lab).

#### Photostability Test by UV Spectrophotometry

Crude extracts, fractions and pure compounds (samples prepared in methanol −100 μg/mL) were subjected to two experimental conditions: exposure to a UVA dose of 27.6 J/cm^2^ emitted by a lamp Philips UVA (Actinic BL/10 lamp – Eindhoven Netherlands) and maintenance in the dark (nonirradiated control). The photostability determination was performed in triplicate. Data analysis was performed by calculating the area under the curve (AUC) of the absorption spectra in the UVB and UVA. Photostability was subsequently assessed as the percentage of photodegradation, calculated from the ratio between the area of irradiated samples and that of the nonirradiated samples.^14^

#### UVB Photoprotection Assay

Prior to any cell-based assay, a cytotoxicity test was performed by using the neutral red uptake (NRU) method to ensure that the observed effects were not due to cell death, according to Rangel et al.^12^ The samples were tested in a range of 50–200 μg/mL and the cytotoxic control used was sodium lauryl sulfate (SDS) at 100 μg/mL.

The UVB photoprotection assay was also performed in HaCaT cells as described by Rangel et al.^12^ HaCaT cells were seeded in two 96-well plates at a density of 1 × 10^5^ cells/well, incubated for 24 h, and then washed with PBS and treated with the samples (50, 100, and 200 μg/mL) and ethylhexyl methoxycinnamate (positive control −100 or 200 μg/mL). One plate maintained in the dark while the other irradiated with a UVB dose of 300 mJ/cm^2^ by a UVB lamp (Broadband TL 40 W/12 RS#, Philips, Netherlands). Untreated and nonirradiated cells were used as negative controls. After irradiation, the cells were washed once, replenished with fresh medium, and incubated for 24 h. The HaCaT cell viability was also obtained by using the NRU method. All assays were carried out in triplicate in three independent experimental runs. The percent of viable cells was calculated using the following equation

$$\%Cell\;Viability=\frac{Absorbance\;sample\;x100}{Absorbance\;untreated\;and\;non-irradiated}$$

#### Photoprotection Potential Against UVA Induced ROS Production in HaCaT Cells

Intracellular ROS produced by HaCaT cell after UVA exposure was evaluated using the probe 2′,7′-dichlorodihydrofluorescein diacetate (DCFH_2_-DA) probe^21^ as previously described by Tavares et al.^20^

HaCaT cells were seeded in a 96-well plate (1 × 10^5^ cells/well) and maintained for 24 h. The cells were then washed with PBS and exposed to the test samples (50, 100, and 200 μg/mL) for 1 h. Quercetin (10 μg/mL) and norfloxacin (100 μg/mL) were used as ROS quencher and generator, respectively. Following treatment, the cells were washed and incubated with DCFH_2_-DA solution (10 μM) for 30 min. After a second wash step, PBS was added to all wells and the plate was irradiated with UVA light at a total dose of 4 J/cm^2^ (Solar simulator Dr. Hönle type SOL-500#, Planegg, Germany).

Immediately after irradiation, the fluorescence was measured using a microplate reader (Synergy HT#, BioTek, USA) at an excitation wavelength of 485 nm and an emission wavelength of 528 nm. All experiments were conducted in triplicate and repeated in three independent assays. The fluorescence of irradiated untreated cells was defined as 100%, and the fluorescence values of the treated groups were expressed relative to this control.

#### Photoprotection Against UVA Induced ROS Production in Reconstructed Human Skin Model

UVA-induced ROS formation was further investigated using reconstructed human skin (RHS) models. All procedures involving primary human keratinocytes and fibroblasts isolated from donated foreskin tissue were approved by the Human Research Ethics Committee of the Faculty of Pharmaceutical Sciences of Ribeirão Preto, University of São Paulo, Brazil (CAAE n° 69358323.9.0000.5403). Written informed consent was obtained from the legal guardians of the donors, and all experimental procedures were conducted in accordance with the ethical principles established in the Declaration of Helsinki.

The RHS models were constructed as described by Tavares et al.^20^ First, the dermal equivalent was prepared by embedding 3.6 × 10^5^ fibroblasts in a type I collagen matrix (Corning, Tewksbury, MA, USA) supplemented with 5% fetal bovine serum (Gibco, Life Technologies, Carlsbad, CA, USA). A total volume of 500 μL was added to each insert of a 24-well plate (Corning, Tewksbury, MA, USA). After 24 h of incubation, 2.9 × 10^6^ primary human keratinocytes were seeded onto the dermis equivalent surface in 2 mL of keratinocyte growth medium. Thus, the culture was maintained at the air–liquid interface for 7 days to promote keratinocyte differentiation.

For ROS quantification, the RHS models were incubated with the DCFH_2_-DA probe diluted in PBS (50 mM) for 45 min at 37 °C in an atmosphere containing 5% of CO_2_. Following PBS washing, the tissues were treated with sescandelin (3) (100 μg/mL) for 1 h. Subsequently, the models were exposed to UVA radiation (10 J/cm^2^), whereas control tissues were maintained in the absence of light. Immediately after the irradiation, the tissues were washed with PBS and frozen using liquid nitrogen. Subsequently, 8 mm cryostat sections were obtained, allowing the fluorescence intensity reading, which was measured using a Ti–S inverted Microscope (Nikon Instruments Inc., Amsterdam Netherlands), 488 nm, at 100 ms exposition intensity. Image analysis was performed by using ImageJ software. Fluorescence values were normalized to the analyzed area (pixels) and expressed as the percentage of fluorescence intensity relative to irradiated and nonirradiated untreated control groups.^20^

### Photosafety Assessment

#### ROS Generation Assay for Photoreactivity (OECD TG 495)

Sample-induced production of singlet oxygen (^1^O_2_, SO) was investigated through monitoring the bleaching of *p*-nitroso-*N*,*N*-dimethylaniline (RNO) at 440 nm by imidazole, which reacts selectively with SO. The formation of superoxide anion (O_2_^–•^, SA) was determined by the measuring the reduction of nitroblue tetrazolium (NBT), monitored as an increase in absorbance at 560 nm, following the OECD TG 495 ROS Assay For Photoreactivity.^22^ All measurements were carried out in triplicate and repeated in two independent runs. Octyl salicylate and histidine (20 μM) were used as negative controls, whereas ketoprofen and quinine (20 μM) were included as positive controls.

#### Phototoxicity Assay (OECD TG 432)

The isolated compounds were evaluated for phototoxicity potential based on neutral red uptake assay using 3T3 murine fibroblasts (BALB/c 3T3 clone A31). The test was performed according to the OECD TG 432 recommendations.^23^

Briefly, the cells (1 × 10^4^) were seeded into two 96-well plates and incubated for 24 h. Then, the fibroblasts were rinsed with PBS with Ca^2+^ and treated with eight sample concentrations (6.74 to 100 μg/mL) for 1 h. Norfloxacin was used as a positive phototoxic control. Subsequently, while one plate was exposed to a UVA irradiation dose of 9 J/cm^2^ using a solar simulator (Dr. Hone type SOL-500#, Planegg, Germany), the other was protected from light. Thus, the plates were rewashed with PBS with Ca^2+^ and a fresh culture medium was added. The plates were then incubated for 24 h and the cell viability was obtained by the NRU assay. The assay was performed in triplicates in two independent experiments. Data was analyzed using Phototox Software 2.0 by calculating the Mean Photo Effect (MPE), a statistical comparison of the dose–response curves obtained with and without UV. According to OECD TG 432 criteria, an MPE <0.10 predicts “no phototoxicity,” while MPE >0.10 and <0.15 predict “probable phototoxicity” and MPE >0.15 predicts “phototoxicity”.

#### Ocular Irritation Potential (HET-CAM Assay)

The Hen’s Egg Choriallantoic Membrane assay (HET-CAM) is a complementary safety test to evaluate the ocular irritation potential and was performed in fertile white Leghorn chicken eggs incubated for 10 days according to the literature described by Luepke and Kemper.^24^ Aliquots of 300 μL of the studied samples were then applied directly onto the CAM surface. A 1% w/w sodium lauryl sulfate solution was used as positive control and 0.9% NaCl solution as the negative control. Sescandelin (**3**) and vermistatin (**4**) were tested at 200 μg/mL (w/v). After 20 s of treatment, the CAM was rinsed with 5 mL of 0.9% NaCl. The irritant effects represented by hyperemia (invisible capillaries become visible, and visible capillaries become redder), hemorrhage (bleeding from the vessels and/or capillaries) and coagulation (opacity on the entire or part of the membrane, or rupture of blood flow in the vessels resulting in a segmented appearance) were monitored for 5 min by stereo microscope (SZT–Led#, BelPhotonics, Brazil). The onset time of each reaction was recorded and converted into individual scores, which were combined to determine the overall irritation score. All analyses were carried out in quadruplicate. According to the Irritation Score obtained, the sample were classified as it follows: between 0 and 0.9, predict a nonirritant substance; between 1.0 and 4.9, predict a slightly irritant substance; from 5.0 to 8.9, predict moderate irritants; and from 9.0 to 21.0, predict severe irritants.^25^

## Results and Discussion

### Identification of Endophytic Fungi

Four fungal strains were isolated from the inner tissues of the Antarctic seaweed *P. antarcticus*. Based on sequencing of the internal transcribed spacer (ITS) region of rDNA, the strains were assigned to *Penicillium* sp., *Cladosporium* sp., *Rhinocladiella similis*, and *Epicoccum* sp. Information regarding isolate identification, sampling site, and the surface-disinfection method used for fungal isolation is summarized in Table 1, while macroscopic and microscopic images of the isolates are provided in Supporting Information (Figure S1).

**Table 1:** Collection Site of *Phaeurus antarcticus*, Surface-Disinfection Methods Used for Fungal Isolation, and Molecular Identification of the Recovered Endophytic Fungi

fungal identification	score	query coverage	*E*-value	identity (%)	genbankaccession number	collection site	disinfection method
*Penicillium* sp. LMC 8102	955	100	0	100%	JN899316.1	Halfmoon Island	MI
*Rhinocladiella similis* LMC 8103	1038	100	0	100%	NR_166008.1	Greenwich Island	MI
*Epicoccum* sp. LMC 8106	793	100	0	99.77%	MH857442.1	King’s George Island	MIII
*Cladosporium* sp. LMC 8108	887	100	0	99.79%	MH864124.1	Greenwich Island	MIII

Previous studies investigating fungi associated with *P. antarcticus* revealed substantial taxonomic diversity and highlighted their potential as a source of bioactive secondary metabolites.^14^ Earlier studies recovered members of the genera *Fusarium*, *Yamadazyma*, *Aspergillus*, *Chaetomium*, *Eurotium*, *Penicillium*, *Arthrinium*, and *Geomyces* genus from this seaweed, with *Aspergillus* and *Penicillium* being the most frequently isolated genera.^14,26^ However, because most of these studies omitted algal surface disinfection of the algal material prior to isolation, the recovered fungi may have included epiphytic taxa, potentially biasing the observed community composition.

To our knowledge, only one previous study has specifically reported the isolation of an endophytic fungus from *P. antarcticus*, namely *Arthrinium* sp., whose secondary metabolites were evaluated for photoprotective activity and structurally characterized.^14^ Moreover, the biotechnological potential explored so far has largely been limited to assays using crude extract, with no detailed chemical profiling or isolation of individual compounds. Thus, the chemical diversity and fungal community associated with this seaweed remain poorly investigated and may represent a promising source of novel compounds with cosmeceutical applications.

### Screening for UVB-UVA Absorbers

The initial stage of the bioprospecting process consisted of screening the crude extracts produced by the isolated fungi to identify samples exhibiting absorption in the UVR region. Therefore, after cultivation and extraction procedures, the crude extracts were submitted to the analysis of their UVR absorption spectra in the UVB and UVA region, and to a photostability evaluation.

Results indicated that the crude extract of *Penicillium* sp. showed the highest absorption profile, mainly in the UVB (280–320 nm) and UVA-II (320–340 nm) regions. *Epicoccum* sp. also presented a promising absorption pattern, although restricted to the UVB range. Regarding photostability, samples showing a reduction greater than 20% in absorbance after UVR exposure are considered photounstable.^20^ Under the evaluated conditions, the extracts of *Cladosporium* sp., *Epicoccum* sp., and *Penicillium* sp. showed satisfactory photostability, whereas the extract of *R. similis* exhibited higher photodegradation in the UVB region (26.1%), indicating lower photostability. A graphical representation of the absorption spectra and the photostability profiles are shown in Figure 1 and Table 2.

**Figure 1:**
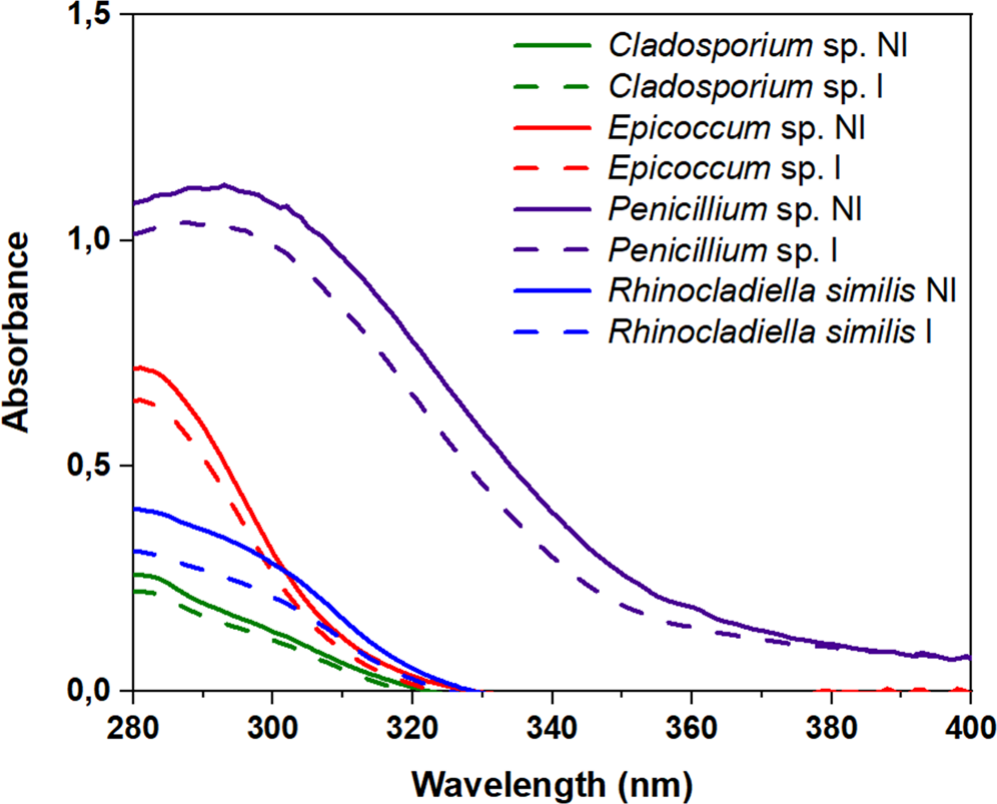
UV absorption spectra of crude extracts (100 μg/mL in MeOH). Photostability was evaluated by comparing the spectra before (solid line) and after (dashed line) UVA irradiation (27.6 J/cm^2^).

**Table 2:** Photodegradation (%) of Crude Extracts, Calculated from the Reduction in the Area Under the UV Absorption Curve of Irradiated Samples Relative to Non-irradiated Samples in the UVA–UVB Region

	percentage of photodegradation relative to nonirradiated pair (%)	
extracts	UVA	UVB
*Cladosporium* sp. (LMC 8108)	-	16.3
*Epicoccum* sp. (LMC 8106)	-	13.0
*Penicillium* sp. (LMC 8102)	18.9	9.3
*R. simillis* (LMC 8103)	-	26.1

Based on the results of the absorbance profile and photostability of its crude extract, the fungus *Penicillium* sp. was chosen for a detailed investigation of its chemical profile and optimization of secondary metabolite production, with the goal of enhancing the production of the photoprotective compounds.

### Optimization of Photoprotective Metabolites Production

#### Chemical Profile by ^1^H NMR

The ^1^H NMR analysis revealed differences in the chemical profiles of extracts obtained from NSW-PDB and ASW-PDB media, indicating that culture medium composition influenced secondary metabolite production (Figure S2). Variations in signal distribution and intensity were observed across cultivation periods, particularly in ASW-PDB derived extracts.^27^ To better visualize the ^1^H NMR data, a PLSDA scatter and loadings plot was constructed and is shown in Figure 3A,B. As observed in the PLSDA scatter plot, samples are separated into three areas and two groups. The pink area represents the extracts from ASW-PDB media, while extracts from NSW-PDB media are represented by the green area. The proximity between samples indicates similarity in their chemical profiles, while greater dispersion indicates higher chemical variability. In the green area, the 14- and 7-days extracts are clustered together in the upper left quadrant, while the 21-days extract is positioned in the bottom right quadrant (Figure 2A). This is indicative that the 7 and 14-days extracts possess similar chemical profiles when compared to the 21-day extract. In the pink area, all the extracts tended to cluster, but the 21-days sample was more dispersed from the 7–14-days extracts, indicating that in the ASW-PDB media, older cultures presented a less similar profile when compared to 7–14-days cultures. In the PLS-DA loadings plot (Figure 2B), it was possible to observe that resonances ranging from 1.00 to 2.00 ppm, characteristics of linear aliphatic chains were the discriminant features in ASW extracts. In the NSW extracts, the discriminant resonances ranged from 2.00 to 4.00 ppm, these shifts are representative of oxygen bearing hydrogens such methoxy and hydroxyl groups and deshielded methyl groups.

**Figure 2:**
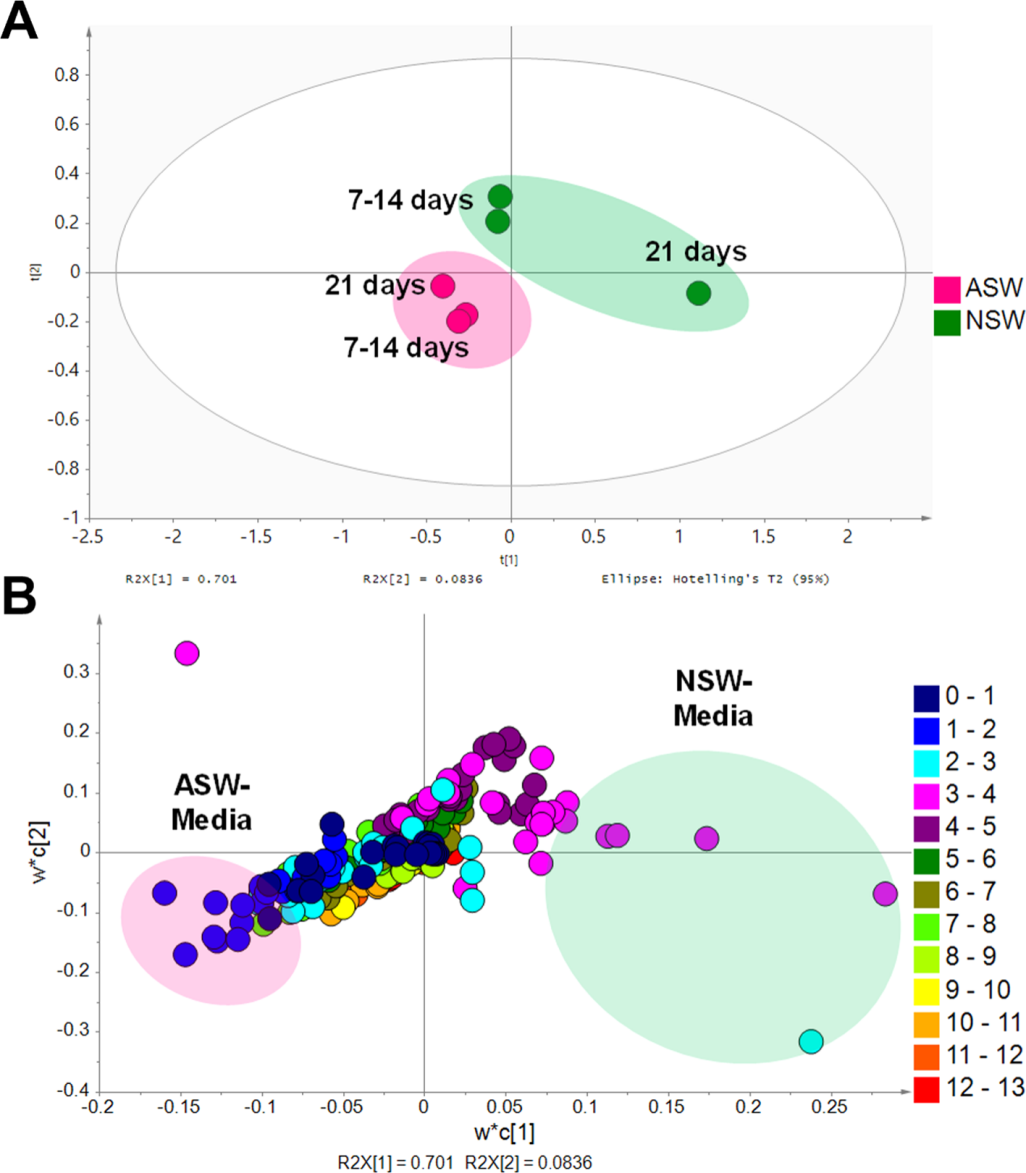
PLS-DA (A) scatter plot of the ^1^H NMR spectral features obtained from *Penicillium* sp. extracts cultivated in ASW-PDB (pink) and NSW-PDB (green) media for 7, 14, and 21 days (*R* ^2^ = 0.99; *Q* ^2^ = 0.90, internal cross-validation), and (B) corresponding loadings plot highlighting the discriminant chemical-shift regions associated with the different cultivation conditions.

**Figure 3:**
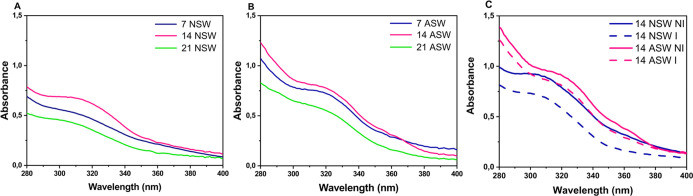
UV absorption spectra of *Penicillium* sp. extracts obtained from (A) NSW-PDB and (B) ASW-PDB media. (C) Photostability assay showing the spectra of 14-day NSW and ASW crude extracts before (solid line) and after (dashed line) UVA irradiation (27.6 J/cm^2^). Representative spectra were obtained from triplicate samples dissolved in MeOH at 100 μg/mL.

#### Determination of the UVR Absorption Spectrum and Photostability of Fungi Crude Extracts

The UV–visible absorption spectra of the EtOAc crude extracts were measured to evaluate how the culture medium and incubation time influence their UVR absorption characteristics to select the optimal conditions for metabolite production. The UVR absorption profiles (Figure 3A,B) showed that, for both media, 14 days of incubation resulted in higher absorption. These findings are consistent with the chemical differences observed in the NMR-based metabolomic analyses and suggest that 14 days of cultivation favored the production of a broader range of UV-absorbing and chemically diverse metabolites, supporting the selection of this incubation period for further analyses. Accordingly, both 14-day crude extracts were submitted to the photostability test (Figure 3C).

Photoprotective compounds typically possess chromophoric moieties that absorb energy within the UV–Vis region. However, irradiation can promote photodegradation processes, reducing their stability and protective capacity. For this reason, photostability assessment is widely used to determine whether UV-absorbing compounds or complex extracts contain photounstable constituents.^14^ Then, the photodegradation percentage between pairs (nonirradiated and irradiated) was determined from the absorption profile after UVA exposure relative to the nonirradiated samples for both 14-day extracts (NSW and ASW). The 14-NSW extract photodegraded 21.3% of its absorbance in the UVB range and 39.1% in the UVA range. In contrast, the 14-ASW extract showed reductions of 9.6% in the UVB range and 14.8% in the UVA range, indicating a more stable profile after irradiation. These findings are important for evaluating the photodegradation characteristics of potential sunscreen ingredients, helping identify stable candidates.

#### Bioguided Isolation of Photoprotective Compounds

Based on the results obtained from the media optimization, *Penicillium* sp. was subjected to a 14-day scale-up fermentation in ASW-PDB medium. After extraction procedures, 7.0 g of the EtOAc extract were subjected to vacuum liquid chromatography (VLC) fractionation, resulting in nine fractions (FrA–FrI). Among them, fractions FrE and FrF were selected for further purification because they presented higher mass yields and exhibited stronger UV absorption profiles, particularly in the UVA region, compared to the remaining fractions. In addition, both fractions were considered photostable after irradiation with a 27.6 J/cm^2^ UVA dose (Table 3 and Figure 4).^20^

**Table 3:** Photodegradation (%) of VLC Fractions Obtained from the ASW-PDB Extract, Calculated From the Reduction in the Area Under the UV Absorption Curve of Irradiated Samples Relative to Non-irradiated Samples in the UVA–UVB Region

percentage of photodegradation relative to nonirradiated pair (%)		
VLC fractions	UVA	UVB
FrE	18.2	5.8
FrF	19.8	10.8

**Figure 4:**
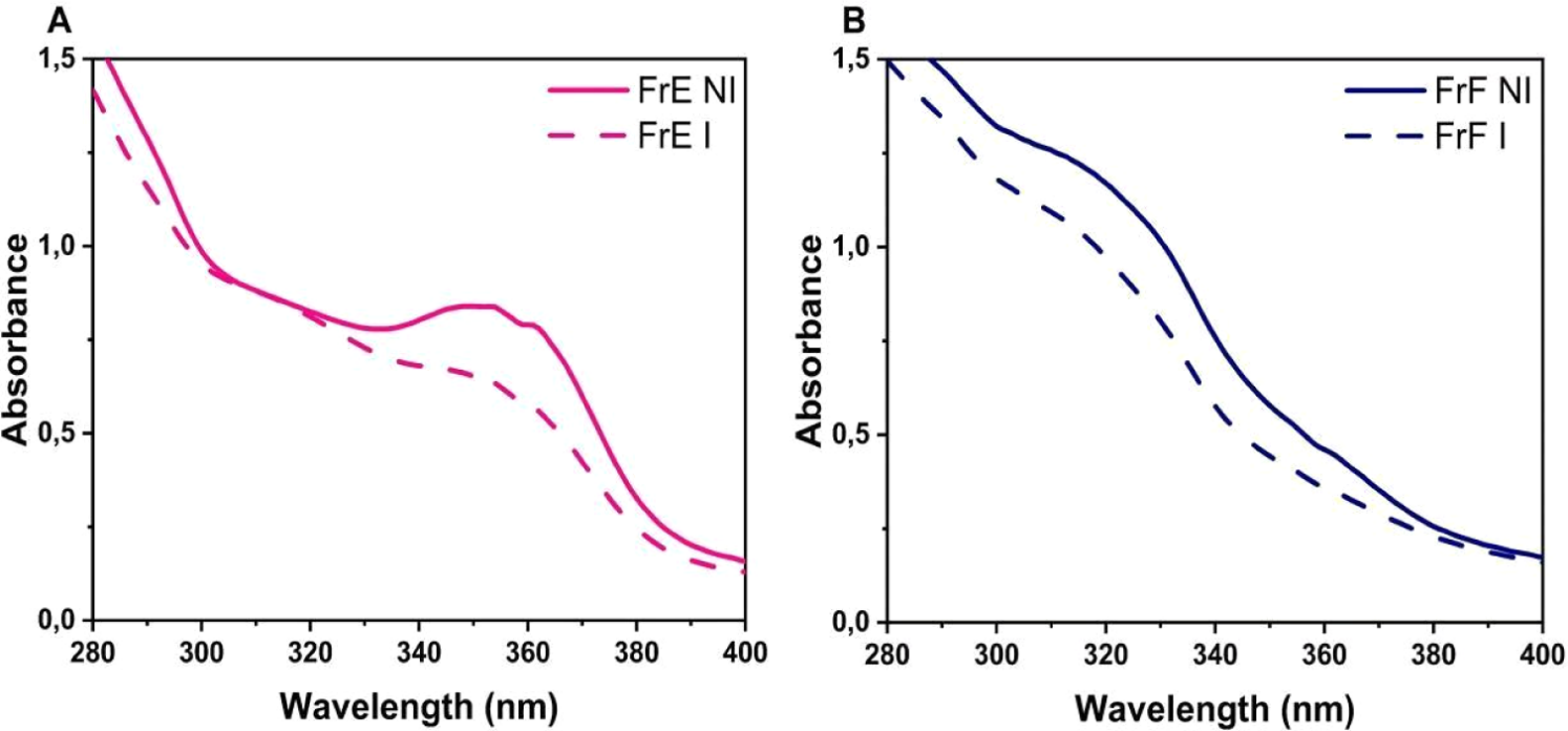
UV absorption profiles of *Penicillium* sp. fractions in the UVA–UVB region: (A) Fraction E and (B) Fraction F. Photostability was evaluated by comparing the spectra before (solid line) and after (dashed line) UVA irradiation (27.6 J/cm^2^). Spectra represent triplicate samples prepared in MeOH at 100 μg/mL.

Current sunscreens often lack sufficient coverage in the UVA range, leaving the skin vulnerable to long-term damage. This limitation is mainly due to the limited number of effective and approved UV filters that operate in this wavelength region, since most available filters predominantly absorb in the UVB range.^5,28^ UVA constitutes the major fraction of terrestrial ultraviolet radiation, accounting for nearly 95% of the UVR reaching the Earth’s surface, and chronic exposure to this radiation has been associated with adverse effects including DNA damage, photoaging, and hyperpigmentation.^4^ Therefore, bioprospecting new filters and substances capable of effectively absorbing this wavelength range is essential. As both fractions showed pronounced absorption in UVA, these fractions were selected for compound isolation.

#### Isolation and Structure Identification of Compounds

The four major compounds were isolated and characterized from FrE and FrF fractions (Figure 5 and 6). The metabolites (1–4) were identified as 5,6,8-trihydroxy-4-(1-hydroxyethyl)-1H-isochromen-1-one (**1**),^29^ aspergillumarin A (**2**),^30^ sescandelin (**3**),^31^ and vermistatin (**4**),^32^ through the interpretation of the HRMS and NMR spectra and comparison with published spectroscopic data.

**Figure 5:**
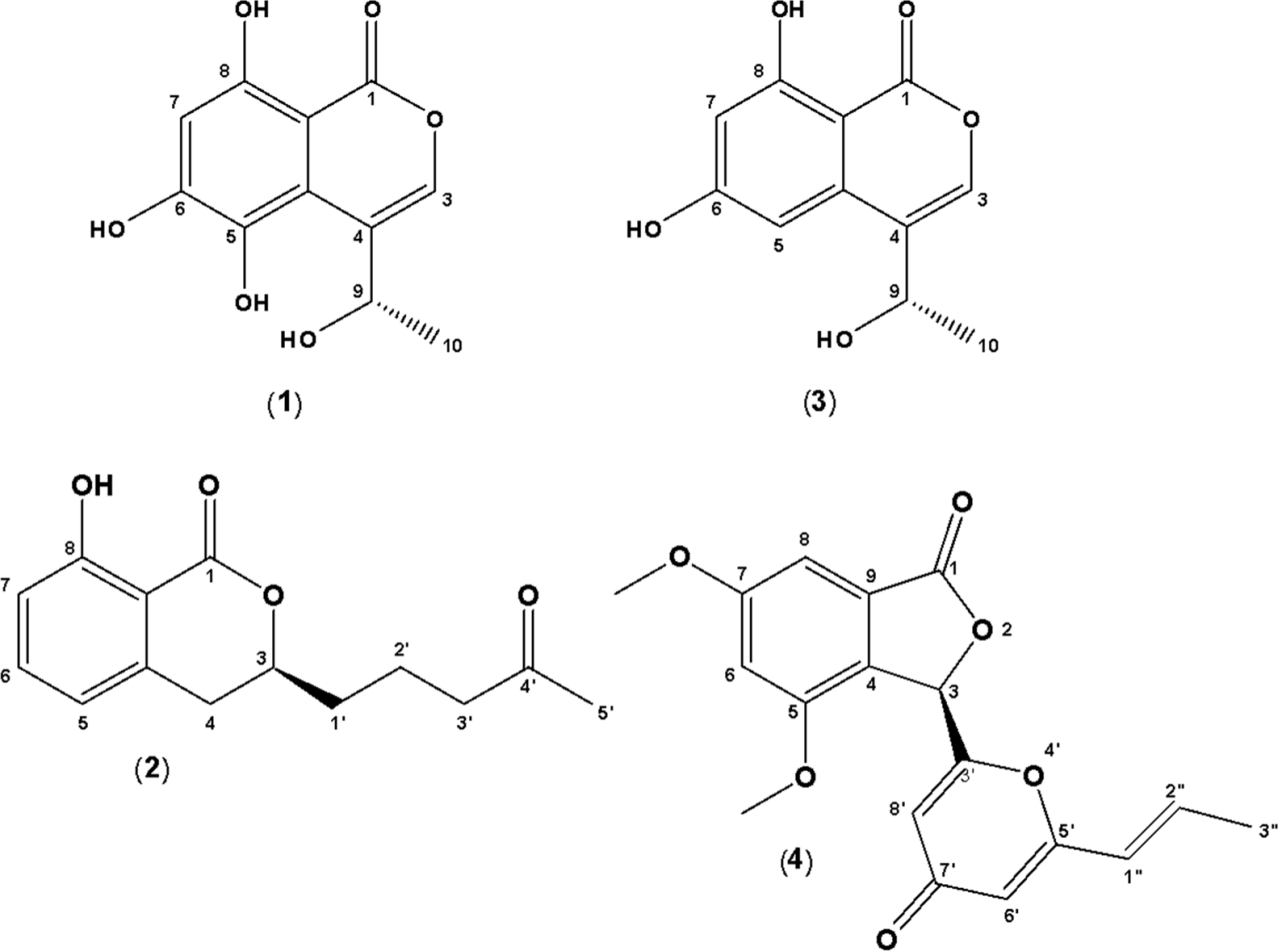
Chemical structures of the major metabolites isolated from FrE and FrF fractions: 5,6,8-trihydroxy-4-(1-hydroxyethyl)-1H-isochromen-1-one (**1**), aspergillumarin A (**2**), sescandelin (**3**) and vermistatin (**4**).

**Figure 6:**
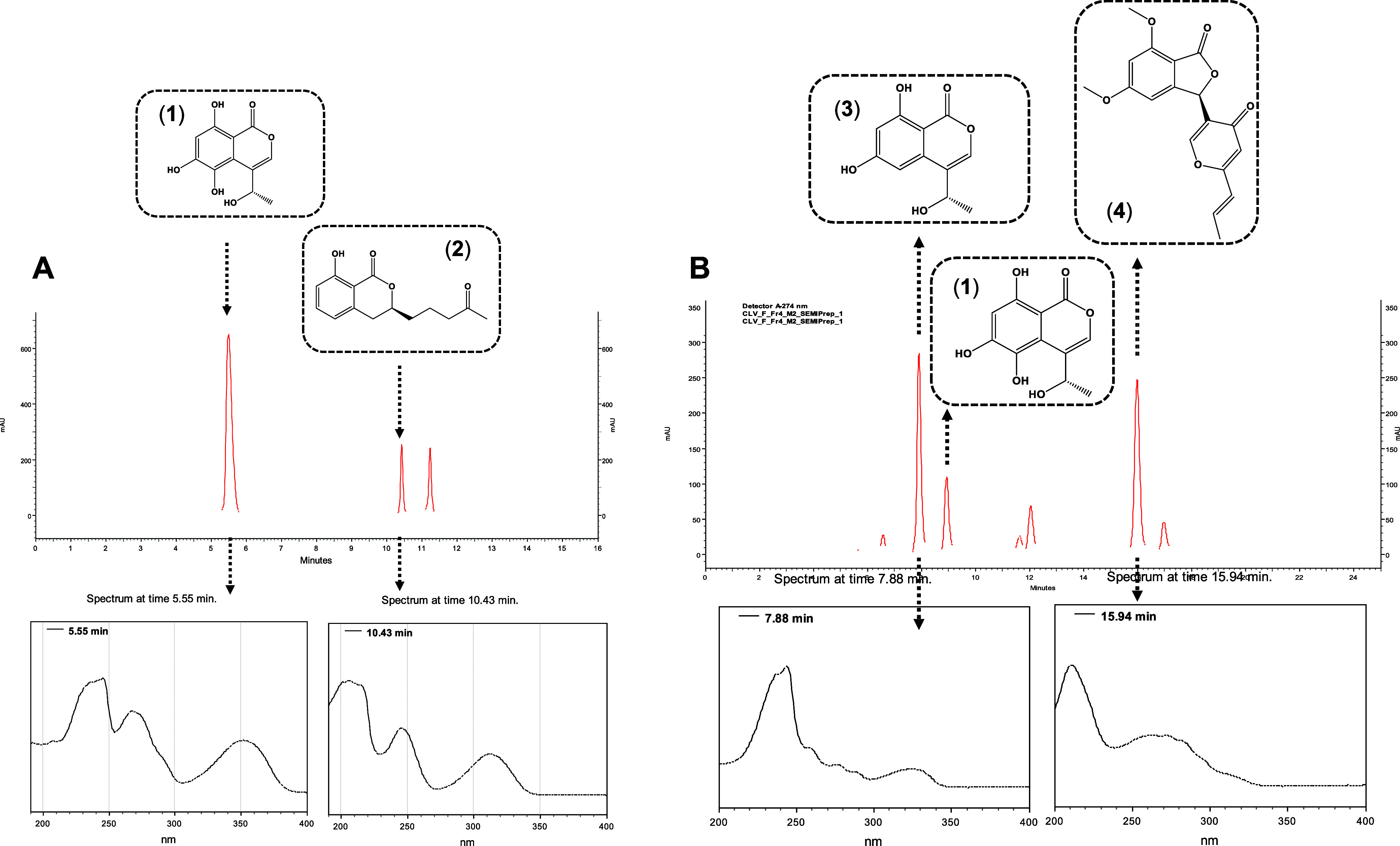
(A) Chromatogram of subfraction FrE-6, elution in a polarity gradient: 0 min −60% H_2_O + 40% CH_3_CN; 7 min −50% H_2_O + 50% CH_3_CN; 10 min −12 min 100% CH_3_CN; 16 min −40% CH_3_CN, monitoring in 329 nm; (B) Chromatogram of subfraction FrF-4, elution in a polarity gradient: 0 min −65% H_2_O + 35% CH_3_CN; 10 min −22 min −25% H_2_O + 75% CH_3_CN; 25 min −65% H_2_O + 35% CH_3_CN, monitoring in 274 nm.

Isocoumarins are a structurally diverse class of fungal polyketides widely distributed among terrestrial and marine-derived fungi and associated with multiple biological activities, including antioxidant, antimicrobial, cytotoxic, and photoprotective effects.^33^ Compound **1** was first reported from an unidentified terrestrial endophytic fungus.^29^ Sescandelin (**3**) was originally isolated from the terrestrial fungus *Sesquicillium candelabrum* and has subsequently been reported from mangrove-derived and marine-associated endophytic fungi, including *Talaromyces amestolkiae* and *Penicillium* sp. TGM112.^31^ Aspergillumarin A (**2**) was first isolated from an endophytic *Aspergillus* sp. associated with the mangrove plant *Bruguiera gymnorrhiza* collected from the South China Sea and later identified in sponge-associated and mangrove-derived fungi, including *Penicillium* spp. and *T. amestolkiae*.^30,34,35^ Vermistatin (4) was initially described from *Talaromyces flavus* (= *Penicillium vermiculatum*) and has also been reported from both terrestrial and marine-derived fungi, including *Pseudocercospora fijiensis* and marine-associated *Penicillium strains*.^36^

Compound **1** (17.0 mg) was isolated as an amorphous white solid, with retention time (Rt) = 5.55 min, U*V*_max_ 246, 238, 351, 207 and U*V*_min_ 239, 254, 208, 202, 197; [α]_D_^26^ – 87 ° (*c*_=_ 0.7 MeOH); High resolution mass spectrometry (HRMS) data presented *m*/*z* 239.0550 [M + H]^+^ corresponding to the molecular formula C_11_H_10_O_6_ (error: 1.2 ppm) (Figure S3). The full structure was determined by ^1^H NMR analyses (Figure S4) (500 MHz, DMSO-*d*_6_, ppm): δ: 7.40 (s) (1H, H-3), 6.50 (s) (1H, H-7), 5.08 (1H, q, *J* = 6.3, H-9), 1.36 (3H, d, *J* = 6.3, H-10) 11.36 (s, OH), 3.36 (s, OH) and ^13^C NMR (500 MHz, DMSO-*d*_6_, ppm): δ: 166.2 (C, C-1), 140.1 (CH, C-3), 119.5 (C, C-4), 119.6 (C, C-4a), 132.1 (C, C-5), 155.9 (C, C-6), 102.7 (CH, C-7), 158.6 (C, C-8), 97.8 (C, C-8a), 67.2 (CH, C-9), 24.0 (CH_3_, C-10) (Figures S5, 6 and Table S1).
Compound **2** (10.0 mg) was isolated as an amorphous solid, corresponding to the peak with Rt = 10.42 min, UV_max_ 206, 201, 213, 246, 312 and UV_min_ 203, 212, 229, 272, 541 nm, [α]_D_^26^ – 89.2° (*c*_=_ 0.8 MeOH); HRMS data presented a *m*/*z* 247.0975 [M – H]^−^, corresponding to the molecular formula C_14_H_16_O_4_ (error: 0.5 ppm) (Figure S7). The ^1^H NMR spectrum presented (500 MHz, CDCl_3_, ppm): δ: 4.58 (1H, m, H-3) 2.94 (2H, m, H-4) 6.69 (1H, d, *J* = 7.4, H-5) 7.41 (1H, dd, *J* = 7.4, 8.4, H-6), 6.89 (1H, d, *J* = 8.4, H-7), 1.84 (2H, m, H-1′), 1.74 (2H, m, H-2′), 2.54 (2H, t, *J* = 6.8, H-3′), 2.16 (3H, s, H-5′), 10.98 (OH, s) (Figure S8) and ^13^C NMR (500 MHz, CDCl_3_, ppm): δ: 173.8 (C, C-1), 79.5 (CH, C-3), 31.9 (CH_2_, C-4), 140.2 (C, C4-a), 117.8 (CH, C-5), 135.6 (CH, C-6), 115.1 (CH, C-7), 161.8 (C, C-8), 107.7 (C, C-8a), 31.9 (CH_2_, C-1′), 18.7 (CH_2_, C-2′), 42.2 (CH_2_, C-3′), 210.0 (C, C-4′), 28.2 (CH_3_, C-5′) (Figures S9, 10 and Table S2).
Compound **3** (12.0 mg), was isolated as a white powder with Rt = 7.88 min. The HRMS analysis revealed an ion peak at *m*/*z* 221.0558 [M – H]^−^ suggesting a molecular formula of C_11_H_10_O_5_ (error: −0.4 ppm) (Figure S11); [α]_D_^26^ −38.3 ° (*c*_=_ 0.9 CHCl_3_). ^1^H NMR of compound **3** (500 MHz, CD_3_OD, ppm) presented similarities with those observed in compound **1**, as follows: δ: 7.38 (1H, br. s, H-3), 6.63 (1H, d, *J* = 2.0, H-5), 6.39 (1H, d, *J* = 2.0, H-7), 4.90 (1H, br. q, *J* = 6.5, H-9), 1.52 (3H, d, *J* = 6.5, H-10), 11.39 (OH, s) (Figure S12) and ^13^C NMR (500 MHz, MEOD4, ppm) δ: 164.7 (C, C-1), 141.7: (CH, C-3), 121.9 (C, C-4), 137.3 (C, C-4a), 102.1 (CH, C-5), 165.3 (C, C-6), 101.3 (CH, C-7), 165.8 (C, C-8), 100.5 (C, C-8a), 63.5 (CH, C-9), 21.7 (CH3, C-10) (Figures S13, 14 and Table S3).
Compound **4** (8.0 mg) was isolated as colorless needles with Rt = 15.96. The HRMS presented an ion peak at *m*/*z* 329.1029 [M-H]^+^ suggesting a molecular formula of C_18_H_16_O_6_ (error: −0.4 ppm) (Figure S15); [α]_D_^26^ −11.3 ° (*c*_=_ 0.3 CHCl_3_). The ^1^H NMR (500 MHz, CDCl_3_, ppm) displayed the following signals δ: 6.46 (1H, s, H-3), 6.68 (1H, d, *J* = 2.1, H-5), 6.98 (1H, d, *J* = 2.1, H-7), 7.42 (1H, s, H-9), 6.16 (1H, s, H-8′), 6.06 (1H, dd, *J* = 15.7, 1.9, H-2″), 6.60 (1H, dq, *J* = 6.9, 15.7, H-1″), 1.92 (3H dd, *J* = 6.9, 1.9, H-3′’), 3.79 (3H, s, H-5), 3.88 (3H, s, H-7) (Figure S16) and ^13^C NMR (500 MHz, CHCl3, ppm) δ: 170.5 (C, C-1), 73.9 (CH, C-3), 127.6 (C, C-3a), 154.7 (C, C-4), 105.2 (CH, C-5), 163.2 (C, C-6), 99.2 (CH, C-7), 127.6 (C, C-7a), 153.9 (CH, C-9), 123.2 (C, C-10), 177.5 (C, C-11), 112.2 (CH, C-12), 161.9 (C, C-13), 123.1 (CH, C-14), 135.8 (CH, C-15), 18.5 (CH_3_, C-16), 56.1 (OMe, C-4), 56.1 (OMe, C-6) (Figures S17, S18, Table S4).

### Photoprotective Potential of *Penicillium* sp. Isolated Compounds

#### UVB/UVA Absorption and Photostability

Compounds 1–4 were isolated and evaluated for their absorption profile and photostability. Compounds were considered photostable, with remaining absorption spectra greater than 80% (Table 4 and Figure 7).^20^ Currently one of the most challenging aspects in the development of new UV filters is photoinstability. Sunscreen stability is essential to maintain photoprotective efficacy, since UV filters may undergo photoisomerization or irreversible photodegradation reactions under UV exposure, leading to loss of activity and potential formation of toxic photoproducts that can damage cells or DNA and affect other formulation components.^37^ In this context, the photostable profile of the metabolites isolated from *Penicillium* sp. reinforces their potential as promising candidates for sunscreen development.

**Table 4:** Remaining UV Absorption (%) After Irradiation, Calculated From the Area Under the UV Absorption Curve of Irradiated Samples Relative to Non-irradiated Samples (100%) in the UVA and UVB Regions

Percentage of remaining absorption relative to the nonirradiated pair (%)		
samples	UVA	UVB
(1)	95.6	89.5
(2)	85.3	93.7
(3)	+17.5	+25.1
(4)	-	+34.4

**Figure 7:**
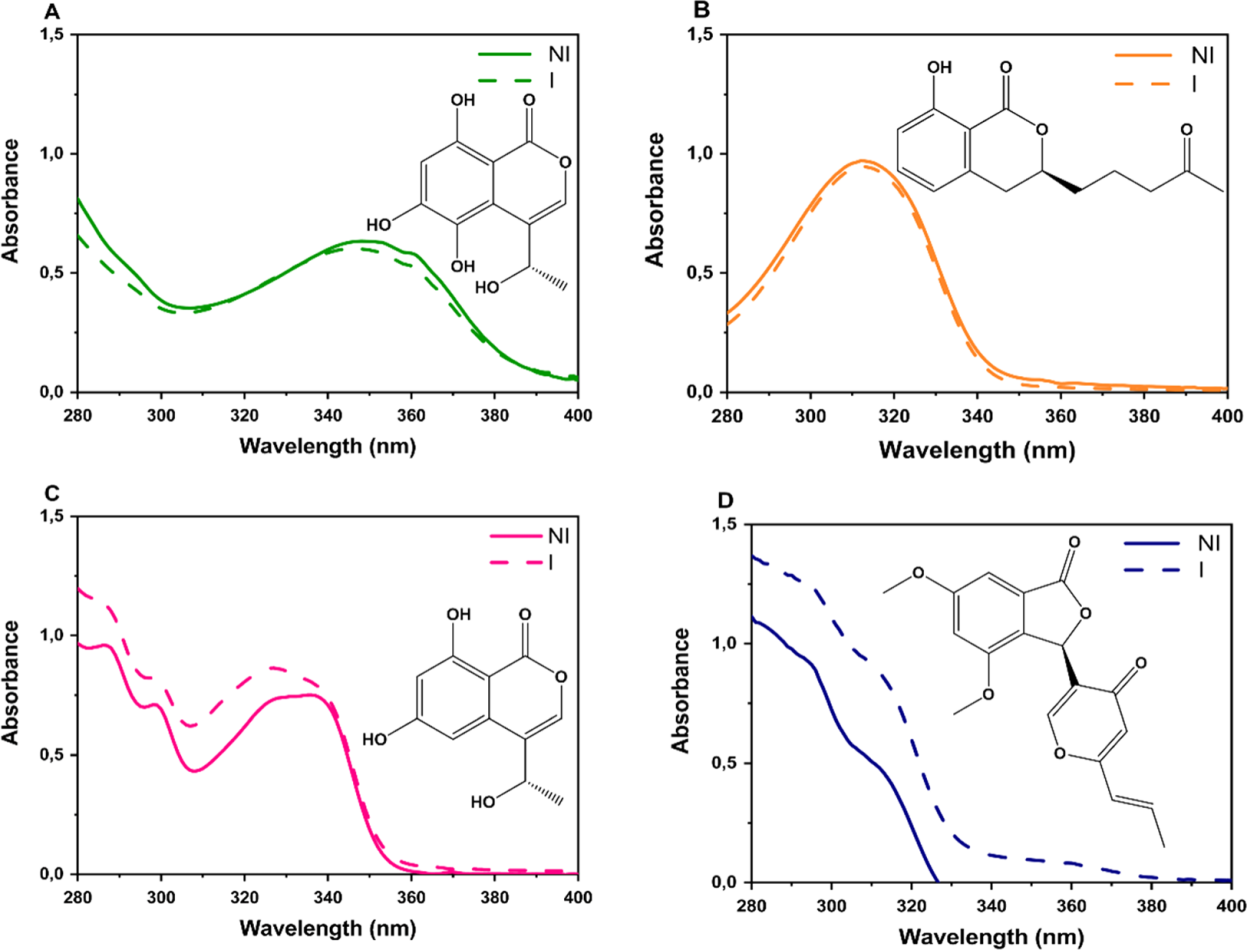
UV absorption spectra and photostability evaluation of the isolated compounds: (A) Compound 1; (B) Compound 2; (C) Compound 3; and (D) Compound 4. Photostability was assessed by comparing the spectra before (solid line) and after (dashed line) UVA irradiation (27.6 J/cm^2^). Compounds 1 and 2 were isolated from fraction FrE, whereas compounds 3 and 4 were obtained from fraction FrF. Representative spectra were obtained from triplicate samples prepared in MeOH at 100 μg/mL.

#### Photoprotective Activity Against UVB

Compounds (2–4), that showed the highest absorption in the UVB region, were selected for the evaluation of photoprotective UVB assay, by using a cell viability test, in which ethylhexyl methoxycinnamate was employed as a reference commercial UV filter.^12,38^ Compound (2) maintained 96% of the cell viability at 200 μg/mL and 64% at 100 μg/mL. Compound (3) also demonstrated UVB protection, with cell viability at 79% (200 μg/mL) and 59% (100 μg/mL). Meanwhile, compound (4) exhibited the highest photoprotection, preserving 100% viability at 200 μg/mL and 94% at 100 μg/mL. Untreated controls showed 32% viability, while MTX-treated cells had 67% viability at both concentrations (Figure 8).

**Figure 8:**
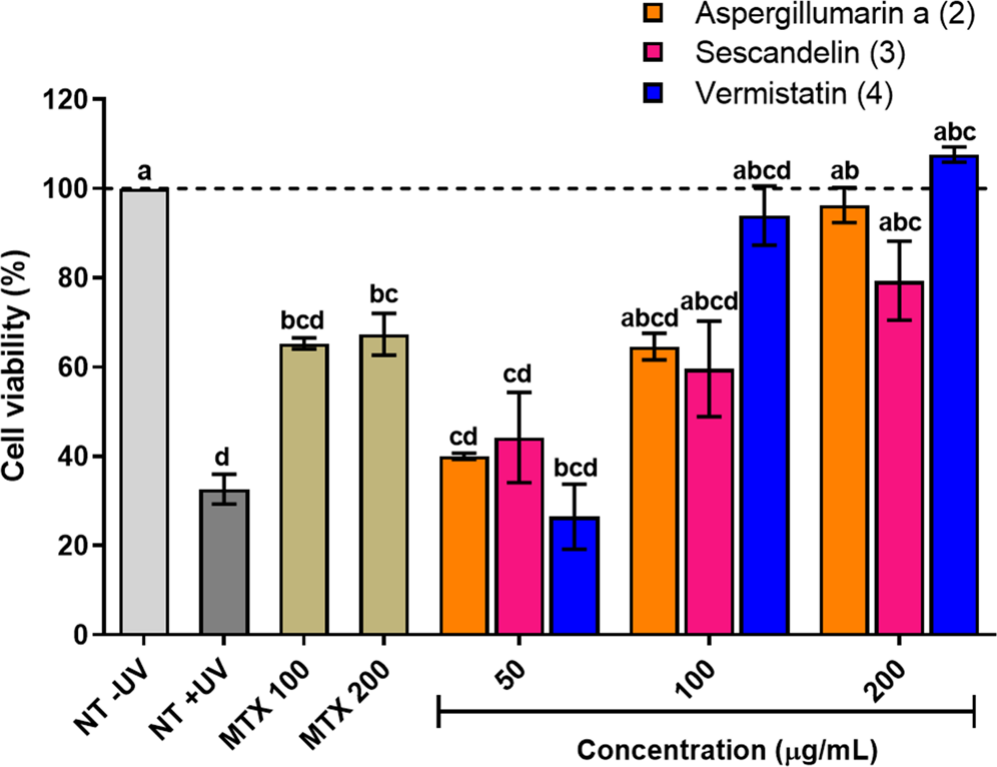
Cell viability of HaCaT cells following treatment with the isolated compounds (200, 100, and 50 μg/mL) or ethylhexyl methoxycinnamate (MTX; 100 and 200 μg/mL) and subsequent UVB irradiation (300 mJ/cm^2^). Untreated nonirradiated (NT – UV) and untreated irradiated (NT + UV) cells were used as controls. Data are expressed as mean ± SEM (*n* = 3).

Coumarins are benzopyrone derivatives reported to have anti-inflammatory, tyrosinase-inhibitory, photoprotective, and antioxidant properties. One example is umbelliferone, a mild natural UV absorber used commercially in sunscreens to support skin photoprotection and reduce hyperpigmentation.^39^ Meanwhile, isocoumarins, such as hydroxymellein from *P. austrosinense*, have shown antioxidant activity and promoted HaCaT cell recovery after UVB exposure.^40^ Components 2 and 3 are also isocoumarins, and despite their potential, these fungal metabolites remain largely underexplored as photoprotective agents.

#### Photoprotection Against UVA-Induced ROS Production in HaCaT Cells

To exclude interference from cytotoxic effects, cell viability was assessed in HaCaT cells prior to the ROS assay. This approach ensured that reductions in fluorescence intensity reflected antioxidant activity. Following ISO 10993-5:2009 criteria, samples with cell viability greater than 70% were considered noncytotoxic.^41^ As shown in Figure 9A, none of the compounds exhibited cytotoxicity at the tested concentrations (50–200 μg/mL).

**Figure 9:**
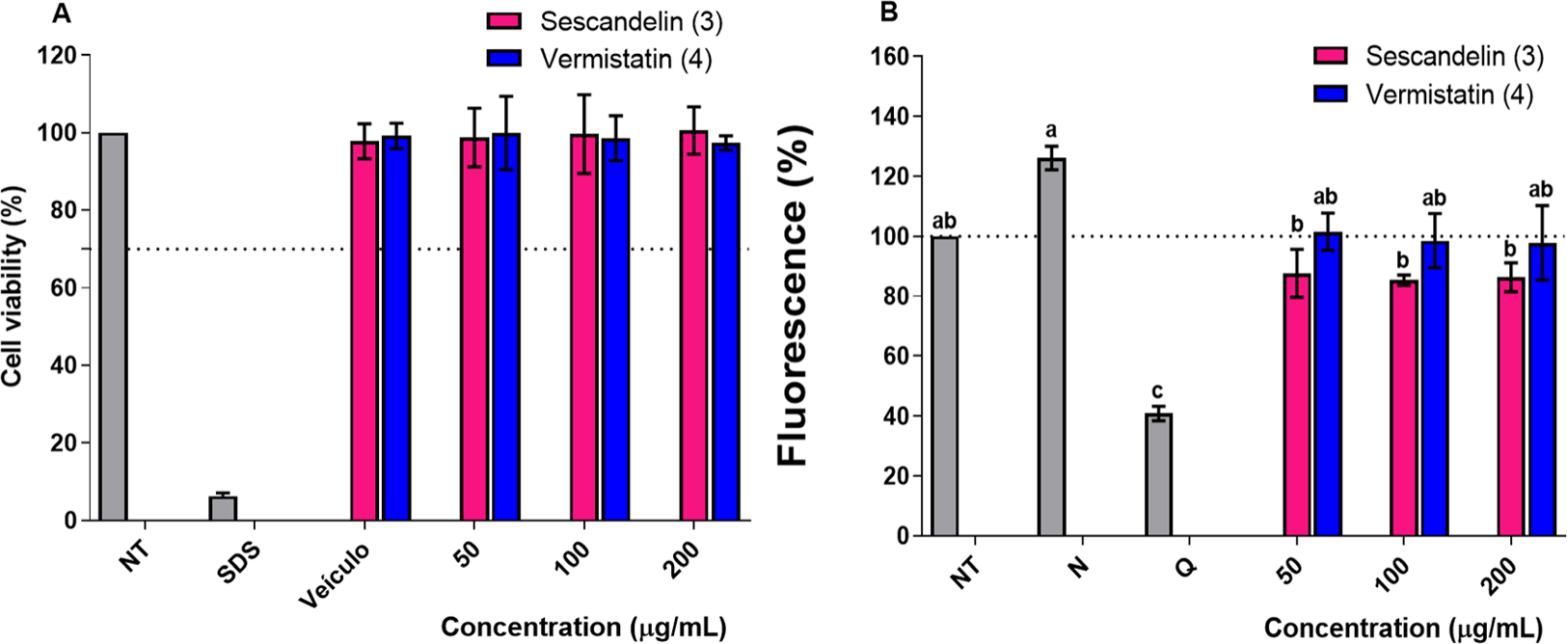
(A) Preliminary cytotoxicity assay in HaCaT cells. (B) Intracellular ROS production induced by UVA irradiation in HaCaT cells after 1 h pretreatment with the test samples. Results are expressed as relative fluorescence (%). Cells were either irradiated (+UV) or nonirradiated (–UV) and treated with quercetin (Q, 10 μg/mL), norfloxacin (N, 100 μg/mL), or compounds 3 and 4 (200, 100, and 50 μg/mL). Different letters denote statistically significant differences among groups (*p* < 0.05).

The fluorescence intensity (%) represents the relative amount of ROS compared with the nontreated control (NT, set as 100%) (Figure 9B). In the antioxidant assay, the results revealed that compound 4 was statistically equal to the NT. Meanwhile, compound 3 was able to attenuate UVA-induced ROS by about 15% at all tested concentrations (200–50 μg/mL); however, no statistically significant difference was observed compared to the nontreated control (NT) (Figure 11B).

**Figure 10:**
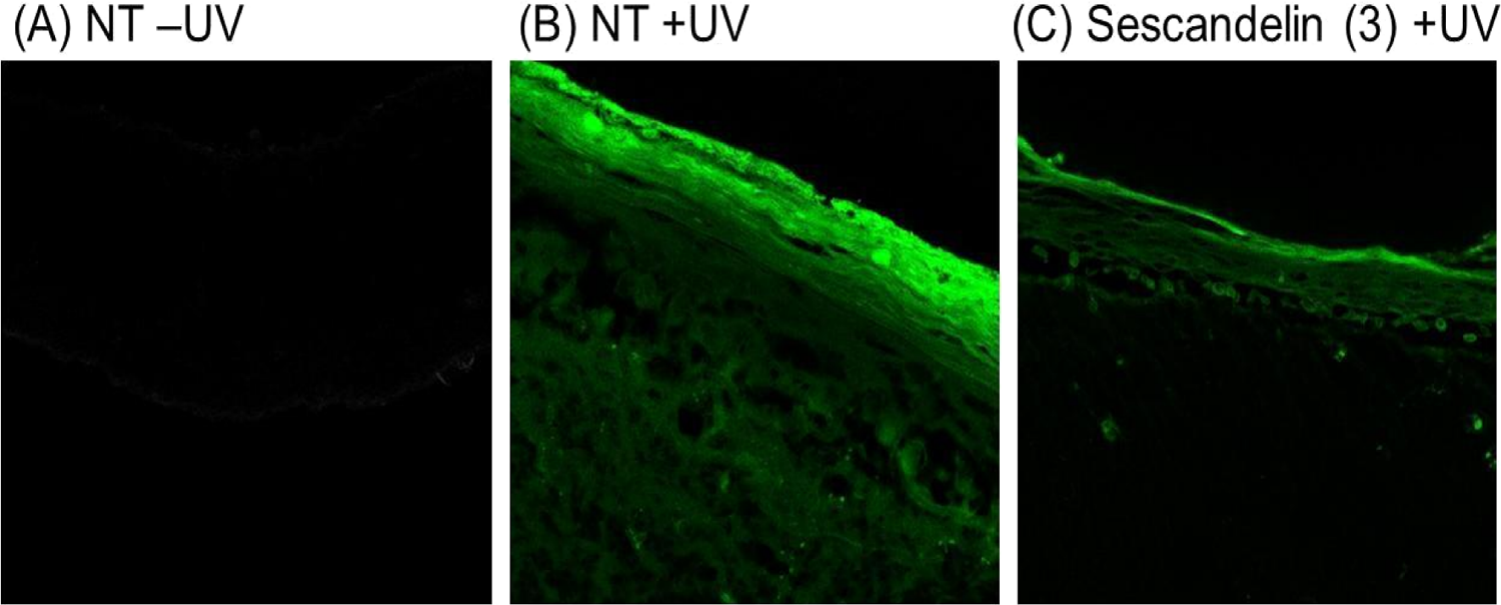
Intracellular ROS levels induced by UVA irradiation in the reconstructed human skin (RHS) model. Results are expressed as the percentage of fluorescent pixels per area. Skin models were (A) untreated and nonirradiated (NT – UV), (B) untreated and irradiated (NT + UV), and (C) treated with Compound 3 (200 μg/mL) formulated in sesame oil (vehicle; 0.02% w/v) prior to UVA exposure.

**Figure 11:**
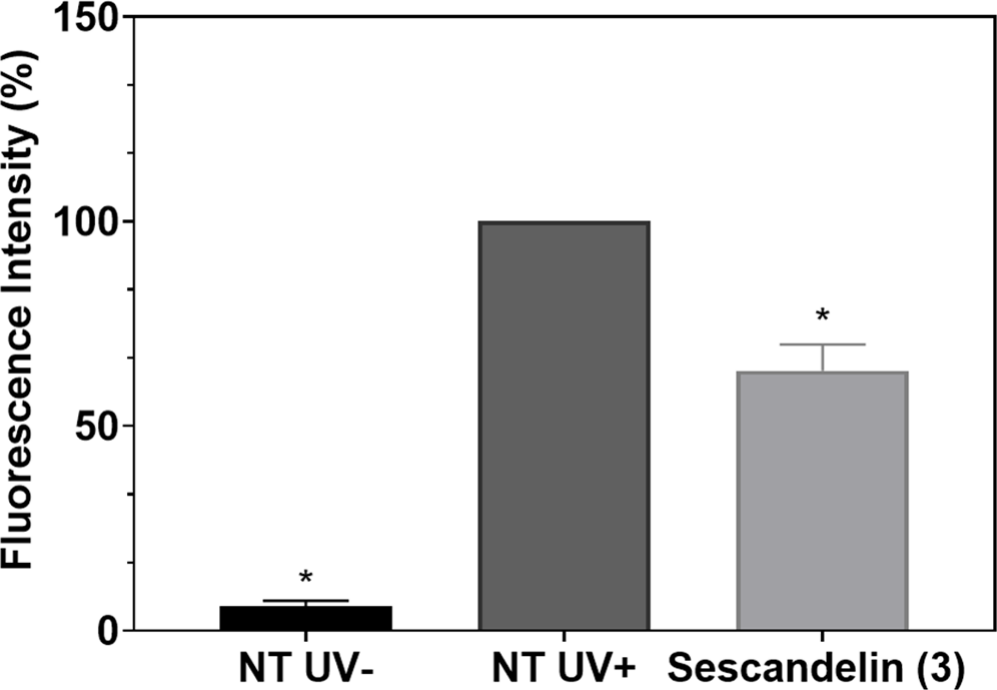
Quantification of intracellular ROS induced by UVA irradiation in the reconstructed human skin (RHS) model using the DCFH_2_-DA probe. Cells were untreated and nonirradiated (NT – UV), untreated and irradiated (NT + UV), or treated with Compound 3 (200 μg/mL) in sesame oil (vehicle; 0.02% w/v) prior to UVA exposure. Fluorescence intensity reflects ROS production. Results are expressed as mean ± SEM from three independent experiments. Statistical significance was determined by one-way ANOVA followed by Tukey’s post hoc test (*p* < 0.05), with asterisks (*) indicating significant differences between groups.

#### Photoprotection Against UVA-Induced ROS Production in RHS

Although compound (3) reduced UVA-induced ROS production in HaCaT cells by approximately 15%, this effect was not statistically significant compared to the nontreated control (NT). Considering the limitations of monolayer cell models (2D) in reproducing the structural and functional complexity of human skin, the photoprotective potential of compound (3) was further evaluated using reconstructed human skin (RHS). The RHS model more effectively engages the skin’s natural antioxidant mechanisms, particularly because it mimics the presence of the stratum corneum, which influences compound penetration.^20^

Compound (3) was able to protect the RHS model from UVA exposure, reducing ROS production by 36.5% compared to the irradiated untreated control (Figures 10 and 11), showing a statistically significant reduction (*p* < 0.05). Similar outcomes were reported for quinoline alkaloids from *P. echinulatum*, which demonstrated stronger antioxidant activity in RHS.^15^

### Photosafety Evaluation

#### Photoreactivity Study – ROS Generation Assay

The photoreactivity potential of the compounds was evaluated by irradiating the samples followed by the measurement of ROS: singlet oxygen (SO) and superoxide anion (SA). Photoreactivity is critical when evaluating UV filters, as ROS generation can lead to oxidative damage and photoaging.^42^ Our findings support the potential of the compounds as nonphotoreactive ingredients (Table 5).

**Table 5:** Photoreactivity Assay of Compounds 2–4. Results are Expressed as Mean ± Standard Deviation From Two Independent Experiments

sample	SO (Δ*A*_440 nm_ × 10^3^)	SA (Δ*A*_560 nm_ × 10^3^)	results^a^
quinine (CP+)	543.50 ± 13.64	336.6 ± 52.87	photoreactive
ketoprofen (CP+)	316.95	101.025	photoreactive
octyl salicylate (CN−)	4.46 ± 8.41	–15.73 ± 1.31	nonphotoreactive
histidine (CN−)	–1.4	17.475	nonphotoreactive
(**2**)	4.76 ± 6.74	–9.10 ± 6.44	nonphotoreactive
(**3**)	4.05	12.725	nonphotoreactive
(**4**)	0.575	14.425	nonphotoreactive

a Photoreactive: SO ≥ 25 and SA ≥ 70, and nonphotoreactive: SO < 25 and SA < 20.

#### Phototoxicity Test (3T3 NRU PT)

In the 3T3 NRU phototoxicity assay, norfloxacin was correctly classified as phototoxic, presenting an MPE value within the range recommended by OECD Test Guideline No. 432.^23,43^ In contrast, among the evaluated metabolites, only compound 3 was classified as nonphototoxic. The results are summarized in Table 6. The 3T3 NRU assay is a standard method for phototoxicity screening; however, positive results require further testing due to its susceptibility to false positives.^44^ The RHS model, which mimics human skin’s barrier, is more predictive of in vivo bioavailability.^15,20^ Viridicatin and viridicatol, isolated from *P. echinulatum*, absorbed UVB and UVA-II radiation and were nonphototoxic in RHS, despite positive results in 3T3 NRU TG.^15^

**Table 6:** Phototoxicity Assay in BALB/C 3T3 Fibroblasts Expressed as MPE Values for Compounds **1**–**4** and the Positive Control (Norfloxacin) From Two Independent Experiments

sample	run	MPE	results
(**1**)	1	0.303	phototoxic
	2	0.157	
(**2**)	1	0.431	phototoxic
	2	0.458	
(**3**)	1	–0.031	nonphototoxic
	2	0.071	
(**4**)	1	0.327	phototoxic
	2	0.307	
norfloxacin	1	0.654	phototoxic
	2	0.507	

#### HET-CAM Eye Irritation Test

The compounds (**3**) and (**4**) received scores of (0 ± 0) and were classified as nonirritants due to the absence of coagulation, hemorrhage, and hyperemia effects. The results for compounds (**3**) and (**4**) are summarized in Table 7 and Figure 12. The HET-CAM assay, evaluates predicts ocular irritation potential using the chorioallantoic membrane of chicken eggs.^24^ This method has been used to assess irritation from fruit extracts,^45^ mycosporine-rich algae,^12^ and polyketides from Antarctic fungi.^14^ The negative control used was 0.9% NaCl and did not present any alterations in the chorioallantoic membrane, being classified as nonirritant (Score = 0 ± 0). Meanwhile, the positive control, SDS, showed hemorrhage and was classified as a severe irritant, as expected (Score = 12 ± 0).^45^

**Table 7:** Irritation Score, Reported as Mean ± Standard Error of the Mean (*n* = 4), and Classification of the Effects of the Compounds in the HET-CAM Assay. Sodium Lauryl Sulfate (SDS) and Sodium Chloride (NaCl) Solutions Were Used as Positive and Negative Controls, Respectively

sample	mean score ±SD	classification
NaCl 0.9%	0 ± 0	nonirritant
SDS 1%	12 ± 0	severe irritant
vehicle (DMSO 1%)	0 ± 0	nonirritant
sescandelin (**3**)	0 ± 0	nonirritant
vermistatin (**4**)	0 ± 0	nonirritant

**Figure 12:**
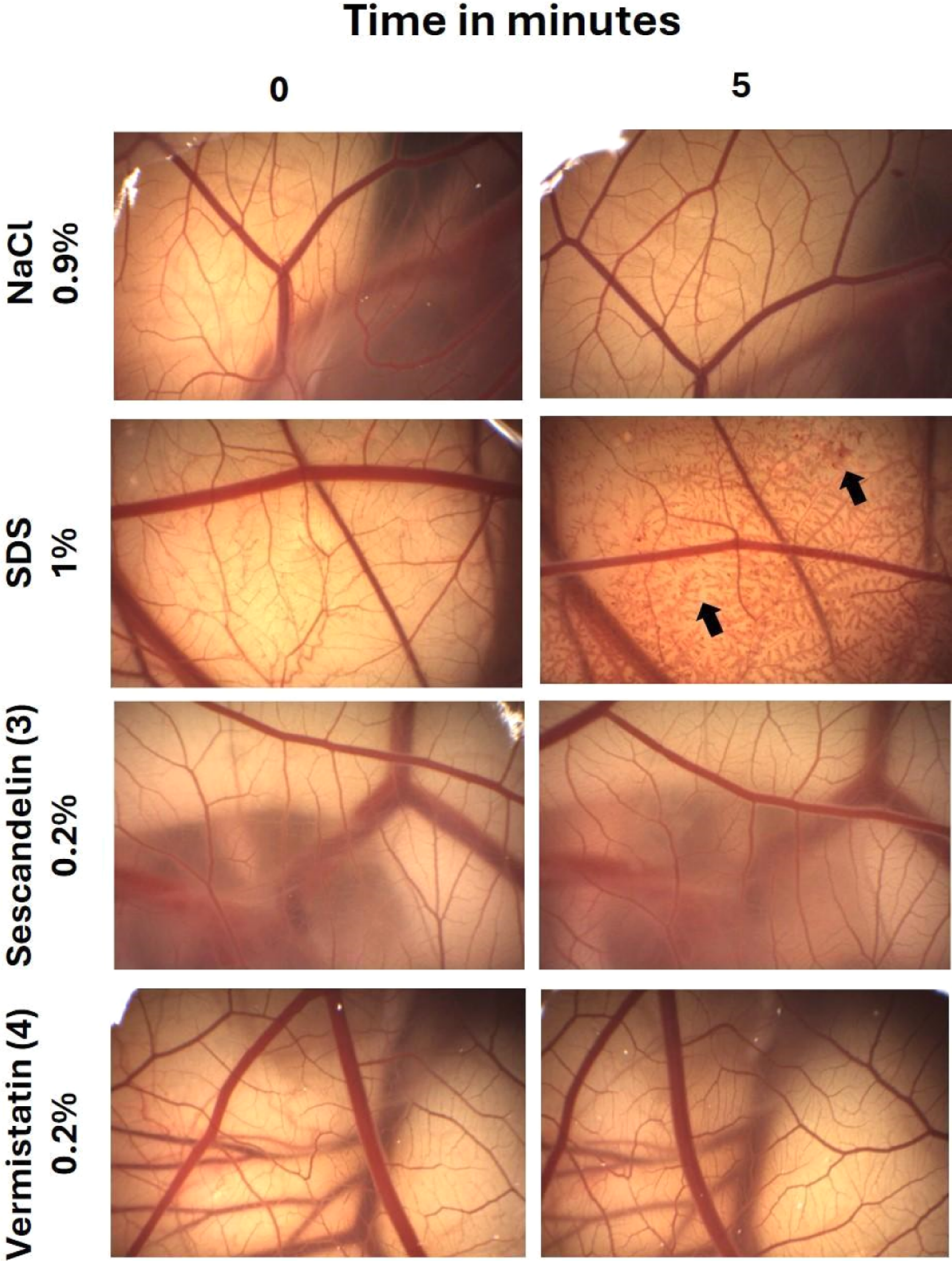
Evaluation of the irritant potential of sescandelin (**3**) and vermistatin (**4**) (0.2%) by the HET-CAM assay. The vascular effects were monitored for 5 min after exposure to each compound. Sodium lauryl sulfate (SDS; 1%) and sodium chloride (NaCl; 0.9%) were used as positive and negative controls, respectively. Black arrows indicate signs of hemorrhage and hyperemia observed in the positive control.

In addition to their photoprotective potential, compounds 2–4 were evaluated against *C. acnes* as a complementary screening assay related to potential dermocosmetic applications (described in Method S1).^46^ However, no antibacterial activity was observed for the tested compounds at 100 μg/mL under the evaluated conditions.

## Conclusion

This study highlights the Antarctic seaweed *P. antarcticus* as a reservoir of endophytic fungi, capable of producing photoprotective secondary metabolites. Through a combination of fungal isolation, molecular identification, culture medium optimization, and bioguided fractionation, four compounds were isolated from *Penicillium* sp. The comprehensive in vitro assessment demonstrated that these compounds exhibit promising UV absorption profiles, photostability, and protective effects against UVB and UVA radiation. Sescandelin (**3**), stood out due to its potent photoprotective and antioxidant activities, combined with a safe profile in our evaluation, reinforcing its potential as a candidate for development in topical sunscreen formulations. These findings contribute to the ongoing search for sustainable, marine-derived photoprotective agents.

## Supplementary Material



## Data Availability

Data are contained within this article or the Supporting Information.

## Notes

Macroscopic and microscopic characterization of fungal isolates, NMR spectra of crude extracts and isolated compounds, HRMS data, and antimicrobial assay method (PDF)
