# Supplementary material for: Bioprospecting of UVB/UVA Absorbers in Fungi Associated with the Macroalga Phaeurus antarcticus Led to the Isolation of Isocoumarins and a Benzofuran with Photoprotective Potential
Source: ACS Omega. 2026 Jul 2;11(28):42681–95. doi: 10.1021/acsomega.6c03690 (PMC13393178; doi:10.1021/acsomega.6c03690)
Supplement: Supplementary file 1 [file ao6c03690_si_001.pdf]

SUPPORTING INFORMATION

Bioprospecting of UVB/UVA absorbers in fungi associated with the macroalga *Phaeurus antarcticus* led to the isolation of isocoumarins and a benzofuran with photoprotective potential

Gustavo Souza dos Santos[a], Karen Cristina Rangel[b], Izadora de Souza[b], Ana Júlia Pasuch Gluzezak[b], Maria Valdeline Sousa Teixeira[b], Ana Carolina Jordão[a], Isadora de Jesus da Silva[b], Ludmilla Tonani[d], Niege Araçari Jacometti Cardoso Furtado[b], Pio Colepicolo[c], Marcia Regina von Zeska Kress[d], RuAngelie Edrada-Ebel[e], Lorena Rigo Gaspar[b], Hosana Maria Debonsi[a]*

[a] Department of Biomolecular Sciences, Faculty of Pharmaceutical Sciences of Ribeirão Preto, University of São Paulo, Prof. Dr. Zeferino Vaz Avenue, Ribeirão Preto, SP 14040-903, Brazil

[b] Department of Pharmaceutical Sciences, Faculty of Pharmaceutical Sciences of Ribeirão Preto, University of São Paulo, Prof. Dr. Zeferino Vaz Avenue, Ribeirão Preto, SP 14040-903, Brazil

[c] Department of Biochemistry, Institute of Chemistry, University of São Paulo, Prof. Lineu Prestes Avenue - 748, São Paulo, SP 05508-000, Brazil

[d] Department of Clinical Analyses, Toxicology and Food Science, Faculty of Pharmaceutical Sciences of Ribeirão Preto, University of São Paulo, Prof. Dr. Zeferino Vaz Avenue, Ribeirão Preto, SP 14040-903, Brazil

[e] Strathclyde Institute of Pharmacy and Biomedical Sciences, University of Strathclyde, 161 Cathedral Street, Glasgow G4 0RE, United Kingdom

* Corresponding author:

E-mail address: hosana@fcfrp.usp.br (H. M. Debonsi)

Figure S1. Macroscopic and microscopic characteristics of fungal endophytes isolated from the Antarctic macroalga *Phaeurus antarcticus*. (A–C) *Penicillium* sp. (LMC 8102); (D–F) *Rhinocladiella similis* (LMC 8103); (G–I) *Epicoccum* sp. (LMC 8106); and (J–L) *Cladosporium* sp. (LMC 8108). Images A, D, G, and J show colony morphology on PDA medium after 14 days of incubation; images B, E, H, and K show the corresponding reverse colony morphology. Images C, F, I, and L show bright-field micrographs obtained at 40× magnification. Scale bars = 20 µm.

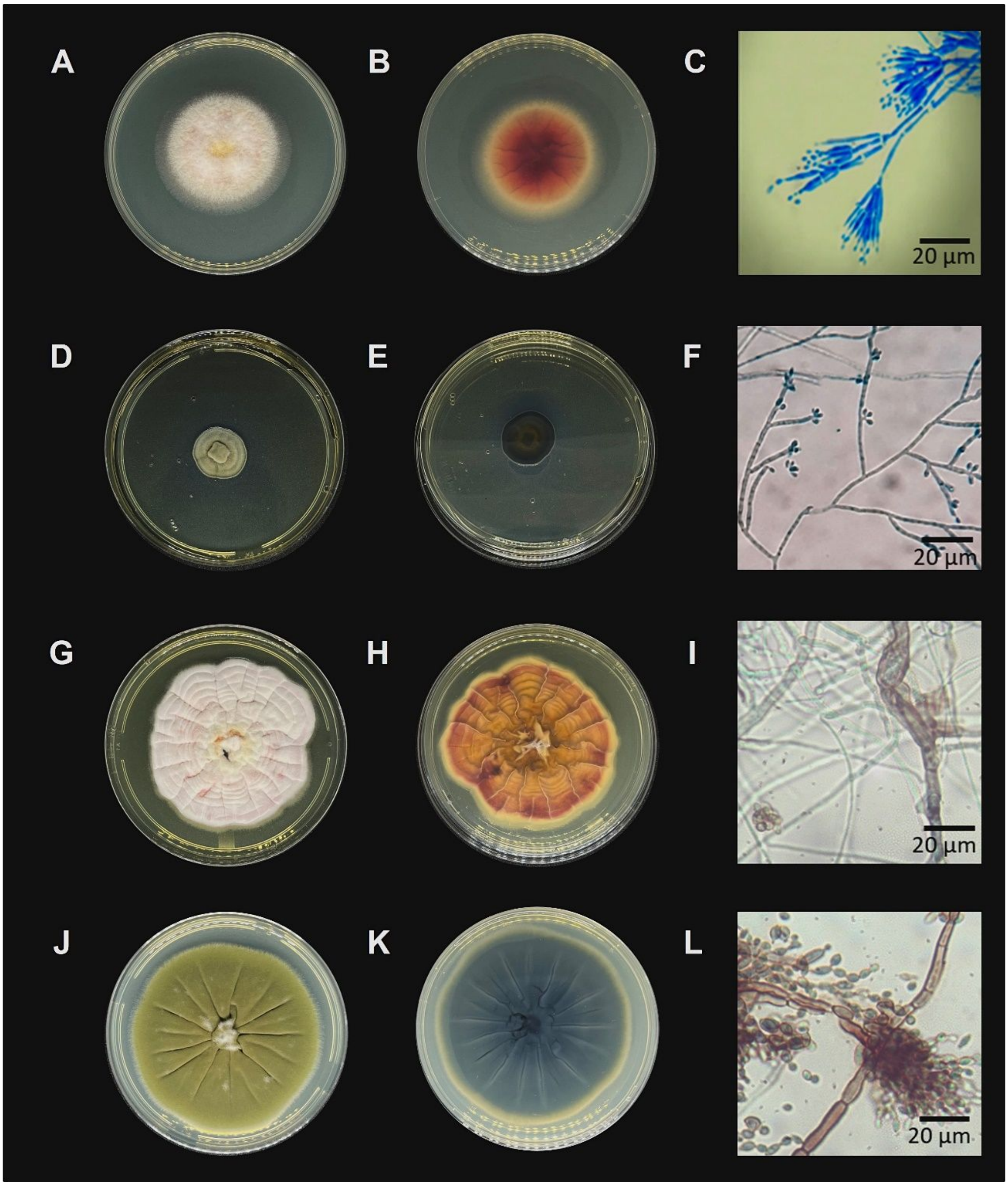

Figure S2. ^{1}H NMR profiles of *Penicillium* sp. crude extracts in DMSO-d_6 (400 MHz).

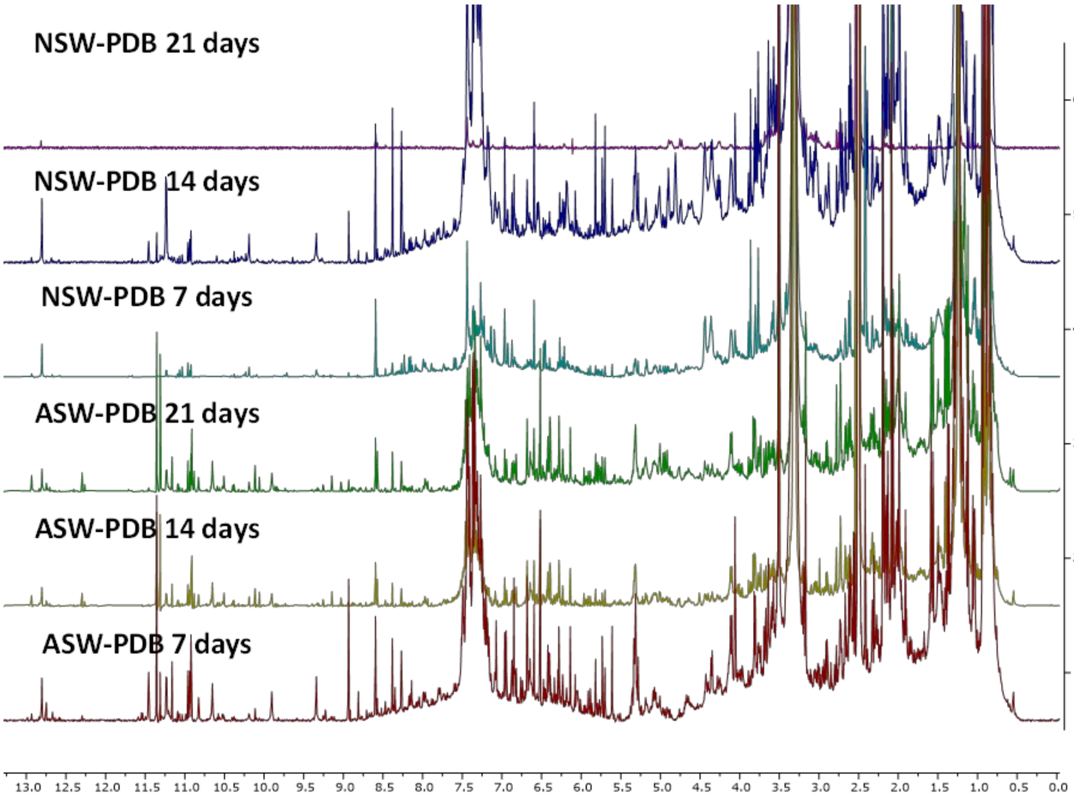

Figure S3. High resolution mass spectrometry of compound **1**

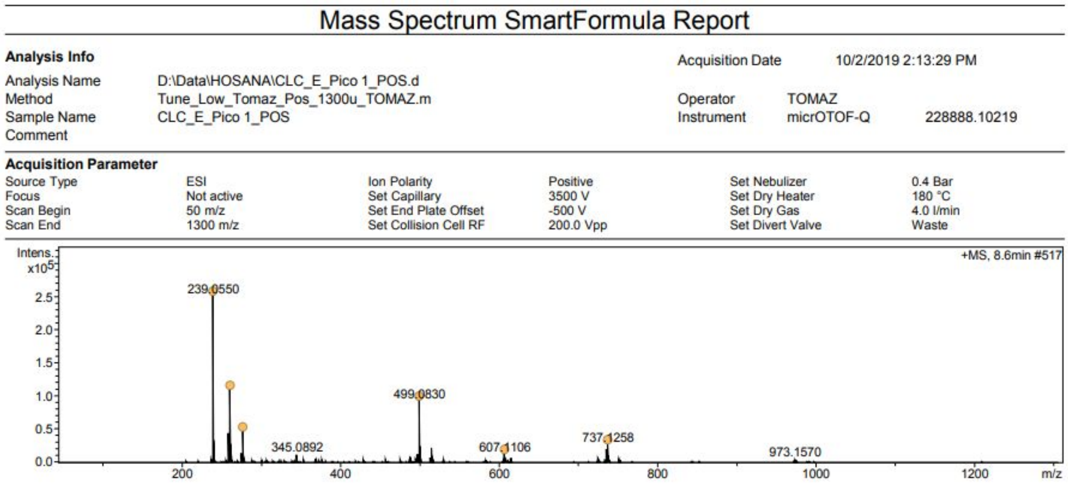

Figure S4. ^{1}H NMR spectra of compound **1** in DMSO-d_6 measured at 500 MHz.

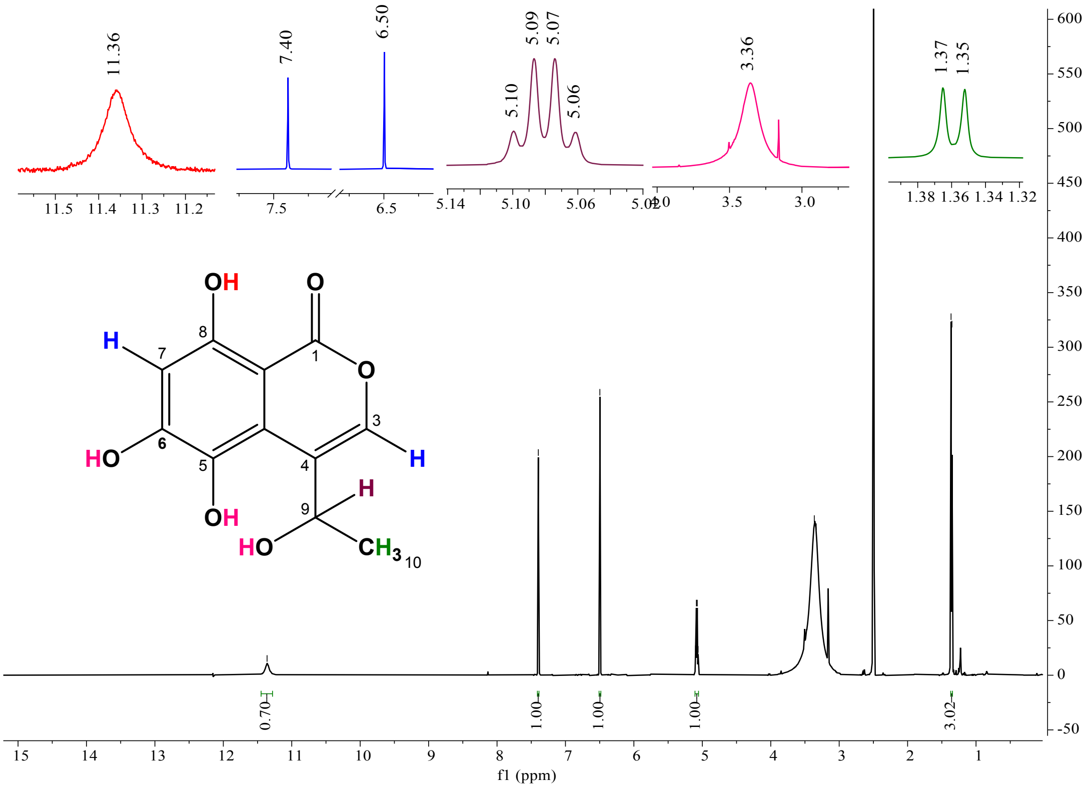

Figure S5. HSQC spectra of compound **1** in DMSO-*d6* measured at 500 MHz.

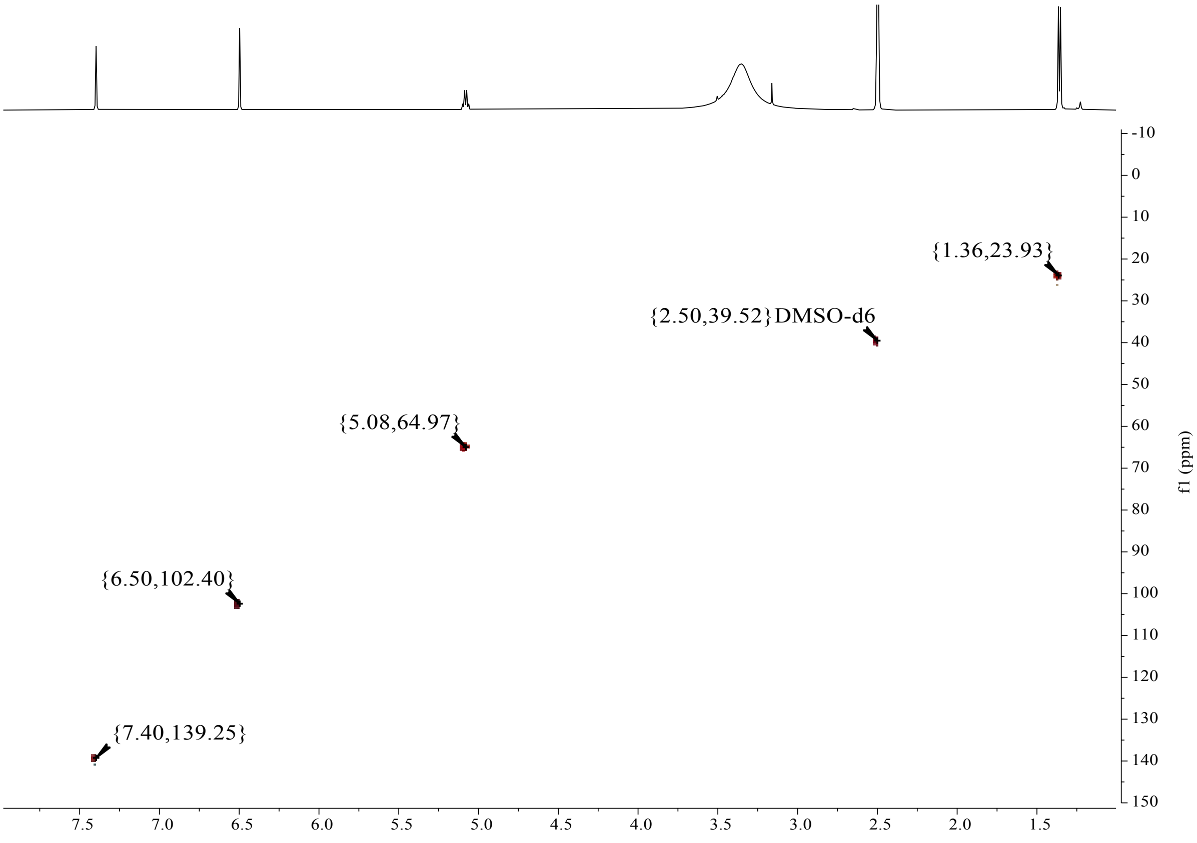

Figure S6. HMBC spectra of compound **1** in DMSO-d_6 measured at 500 MHz.

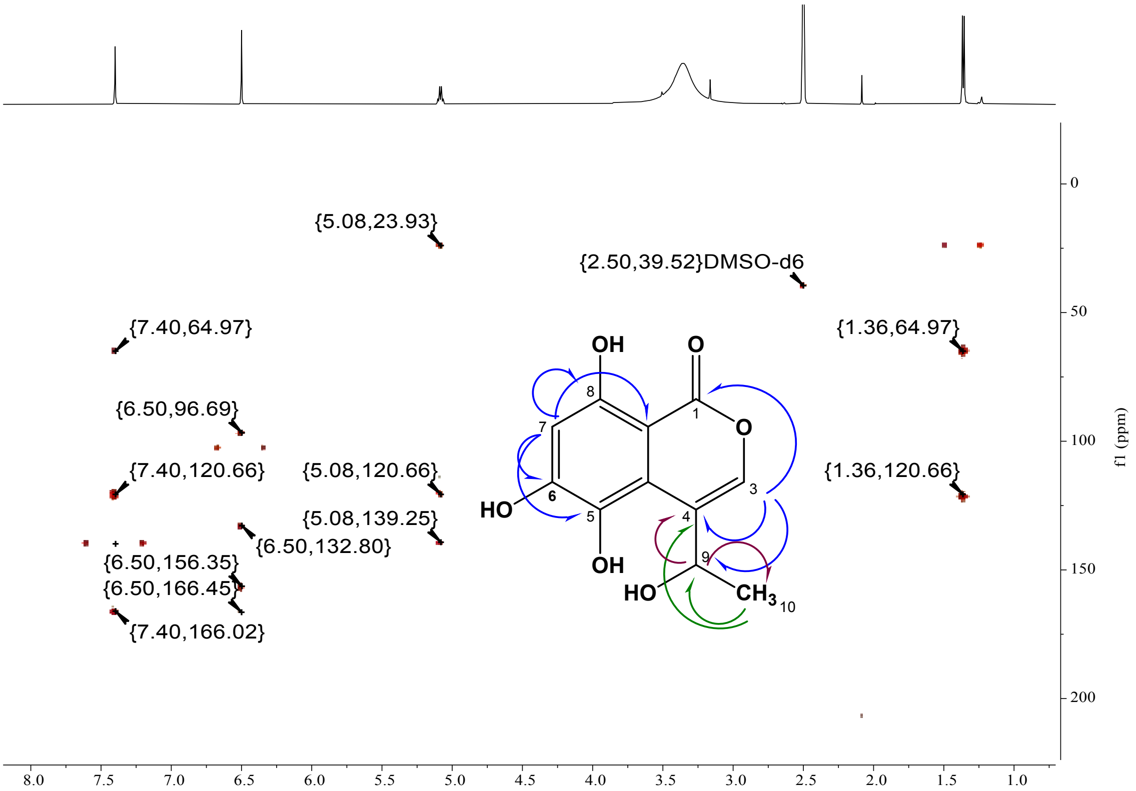

Table S1. ^{1}H and ^{13}C NMR experimental data of compound 1 in comparation to literature data of 5,6,8- trihydroxy-4-(1′-hydroxyethyl) isocoumarin.

	5,6,8- trihydroxy-4-(1′-hydroxyethyl) isocoumarin 400 MHz, acetone-d^6 (FINDLAY et al., 2003)		Compound **1** 500 MHz, DMSO-d_6 (Experimental)	
	**^{13}C δ ppm**	**^{1}H – δ ppm (mult, *J Hz*)**	**^{13}C δ ppm**	**^{1}H – δ ppm (mult, *J Hz*)**
**C-1**	166.2	-	166.0	-
**C-3**	140.1	7.36 (s)	139.2	7.40 (s)
**C-4**	119.5	-	120.6	-
**C-4a**	119.6	-	120.6	-
**C-5**	132.1	-	132.8	-
**C-6**	155.9	-	156.3	-
**C-7**	102.7	6.56 (s)	102.5	6.50 (s)
**C-8**	158.6	-	156.3	-
**C-8a**	97.8	-	97.2	-
**C-9**	67.2	5.02 (q, 6.7)	64.9	5.08 (q, 6.3)
**C-10**	24.0	1.54 (d, 6.7)	23.9	1.36 (d, 6.3)
**8-OH**	-	11.47 (s)	-	11.36 (s)
**9-OH**	-	6.05 (bs) [a]	-	3.36 (bs)

Figure S7. High resolution mass spectrometry of compound **2**

Mass Spectrum SmartFormula Report

Analysis Info

Analysis Name	D:\Data\Profa HOSANA\CLV_E_Pico 3_Fr 6_NEG.d	Acquisition Date	12/19/2018 3:28:14 PM
Method	tune_low_DANIEL 1300 daltons_NEG.m	Operator	BD@AL
Sample Name	CLV_E_Pico 3_Fr 6_NEG	Instrument	micrOTOF 213750.10361
Comment			

Acquisition Parameter

Source Type	ESI	Ion Polarity	Negative	Set Nebulizer	0.4 Bar
Focus	Not active			Set Dry Heater	180 °C
Scan Begin	50 m/z	Set Capillary	3500 V	Set Dry Gas	4.0 l/min
Scan End	1300 m/z	Set End Plate Offset	-500 V	Set Divert Valve	Waste

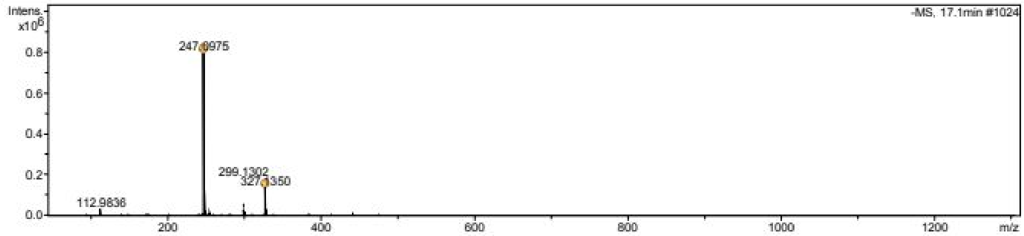

Meas. m/z	#	Ion Formula	m/z	err [ppm]	mSigma	# mSigma	Score	rdb	e⁻ Conf	N-Rule
247.0975	1	C14H15O4	247.0976	0.5	1.0	1	100.00	7.5	even	ok
327.1350	1	C19H15N6	327.1364	4.1	13.1	1	62.48	15.5	even	ok
	2	C18H19N2O4	327.1350	0.0	23.4	2	100.00	10.5	even	ok

Figure S8. ^{1}H NMR spectra of compound **2** in $CDCl_3$ measured at 500 MHz.

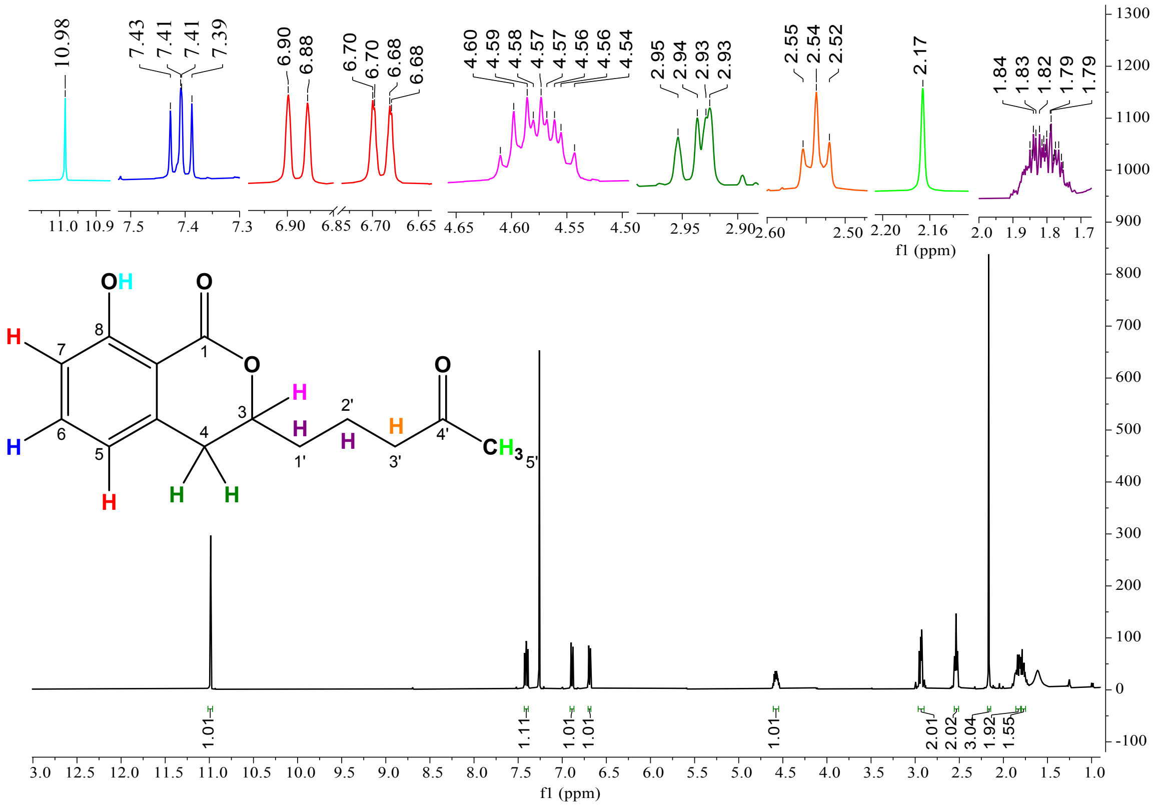

Figure S9. HSQC spectrum of compound **2** in $CDCl_3$ measured at 500 MHz.

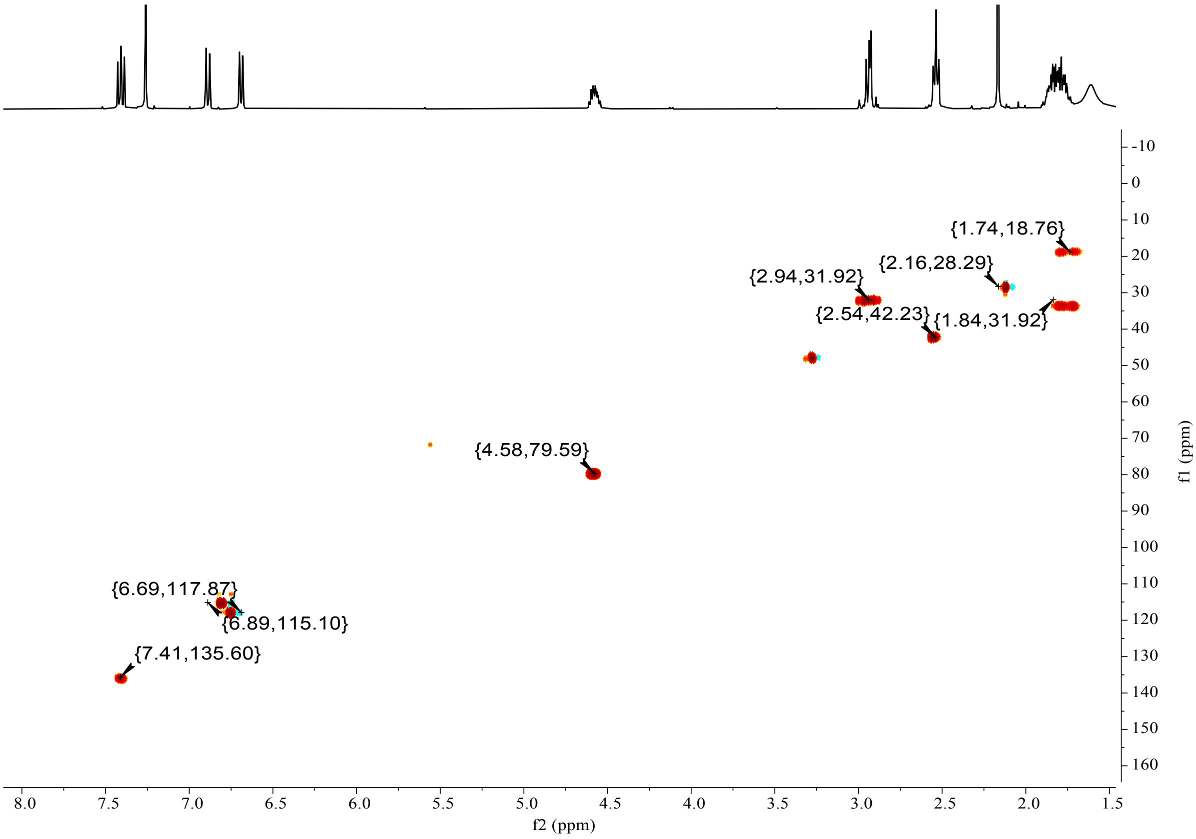

Figure S10. HMBC spectrum of compound **2** in $CDCl_3$ measured at 500 MHz.

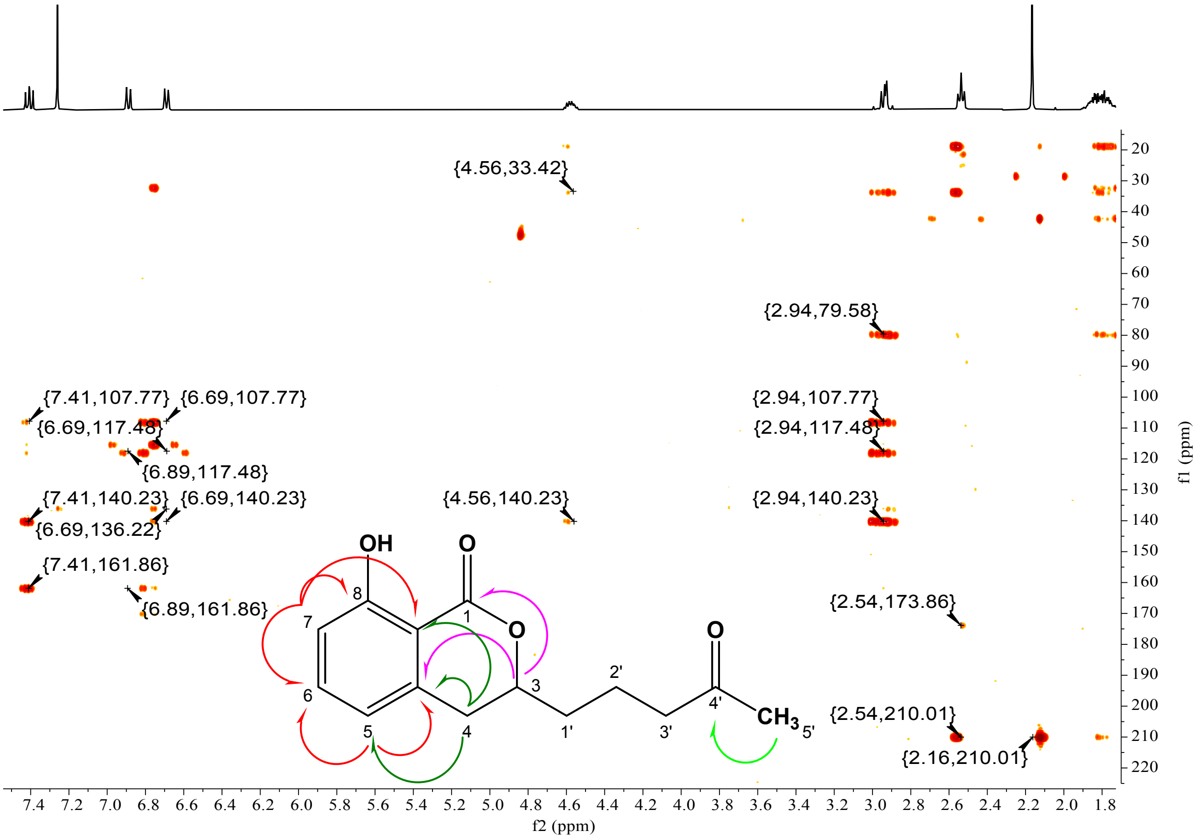

Table S2. ^{1}H and ^{13}C NMR experimental data of compound **2** in comparation to literature data of aspergillumarin A.

	Aspergillumarin a<br>500 MHz, $CDCl_3$<br>(LI et al., 2012)		Compound **2**<br>500 MHz, $CDCl_3$<br>(Experimental.)	
	**^{13}C δ ppm**	**^{1}H – δ ppm (mult, *J Hz*)**	**^{13}C δ ppm**	**^{1}H – δ ppm (mult, *J Hz*)**
**C-1**	169.7	-	173.8	-
**C-3**	79.3	4.57 (m)	79.5	4.58 (m)
**C-4**	33.9	2.92 (br. s)	31.9	2.94 (m)
**C-4a**	139.2	-	140.2	-
**C-5**	117.9	6.69 (d, 7.2)	117.8	6.69 (d, 7.4)
**C-6**	136.1	7.40 (dd, 8.4, 7.2)	135.6	7.41 (dd, 9.4)
**C-7**	116.0	6.87 (d, 8.4)	115.1	6.89 (d, 8.4)
**C-8**	162.0	-	161.8	-
**C-8a**	108.2	-	107.7	-
**C-1’**	33.9	1.83 (m)	31.9	1.84 (m)
**C-2’**	18.9	1.76 (m)	18.7	1.74 (m)
**C-3’**	42.7	2.53 (t, 6.8)	42.2	2.54 (t, 6.8)
**C-4’**	208.2	-	210.0	-
**C-5’**	29.8	2.16 (s)	28.2	2.16 (s)
**8-OH**	-	10.97 (s)	-	10.98 (s)

Figure S11. High resolution mass spectrometry of compound **3**

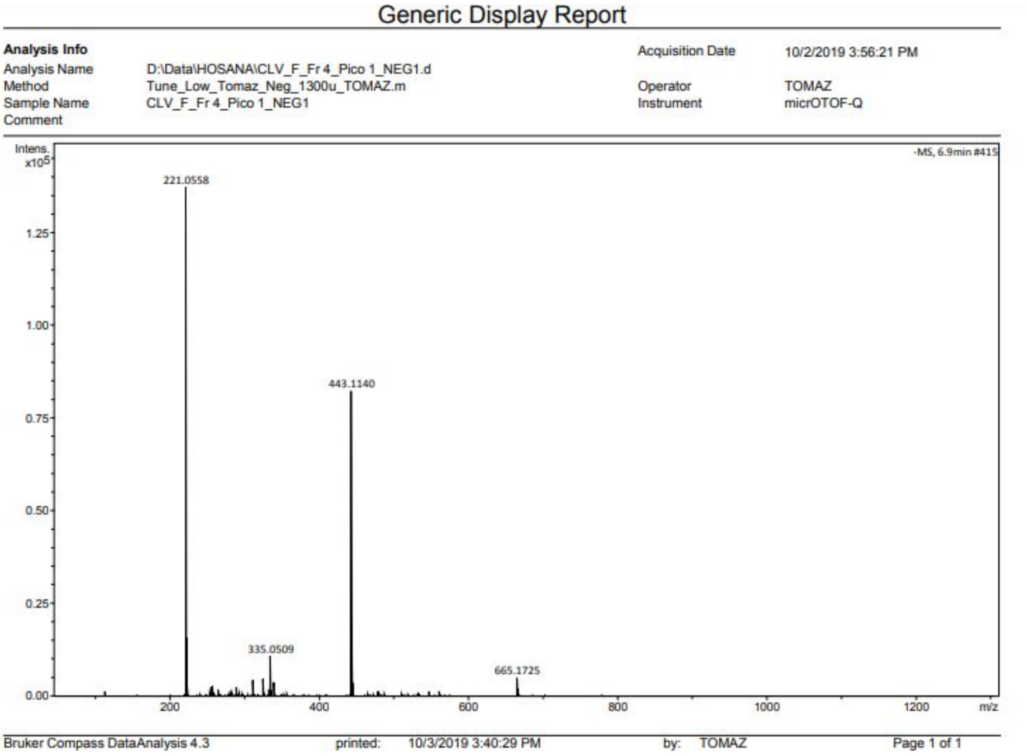

Figure S12. ^{1}H NMR spectrum of compound **3** in $MeOD_4$ (500 MHz) .

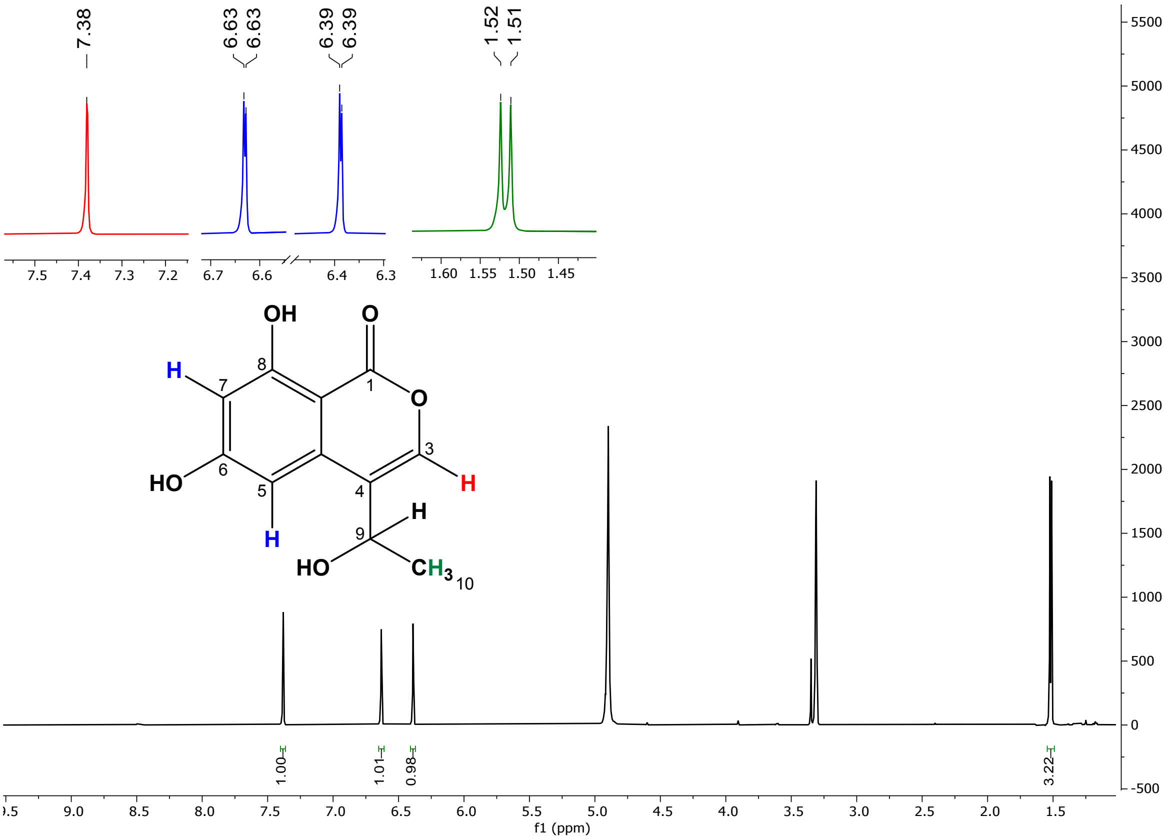

Figure S13. HSQC correlation spectrum of compound **3** in $MeOD_4$ (500 MHz).

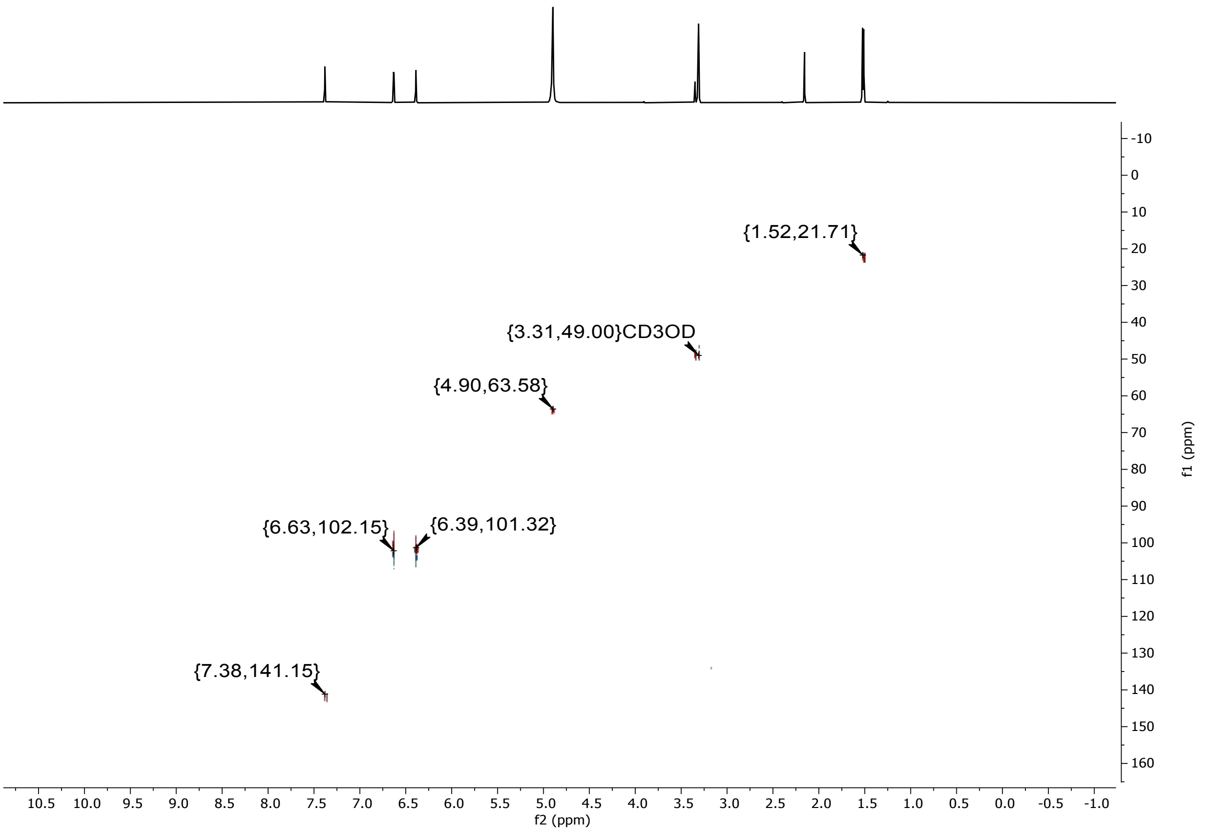

Figure S14. HMBC correlation spectrum of compound **3** in $MeOD_4$ (500 MHz).

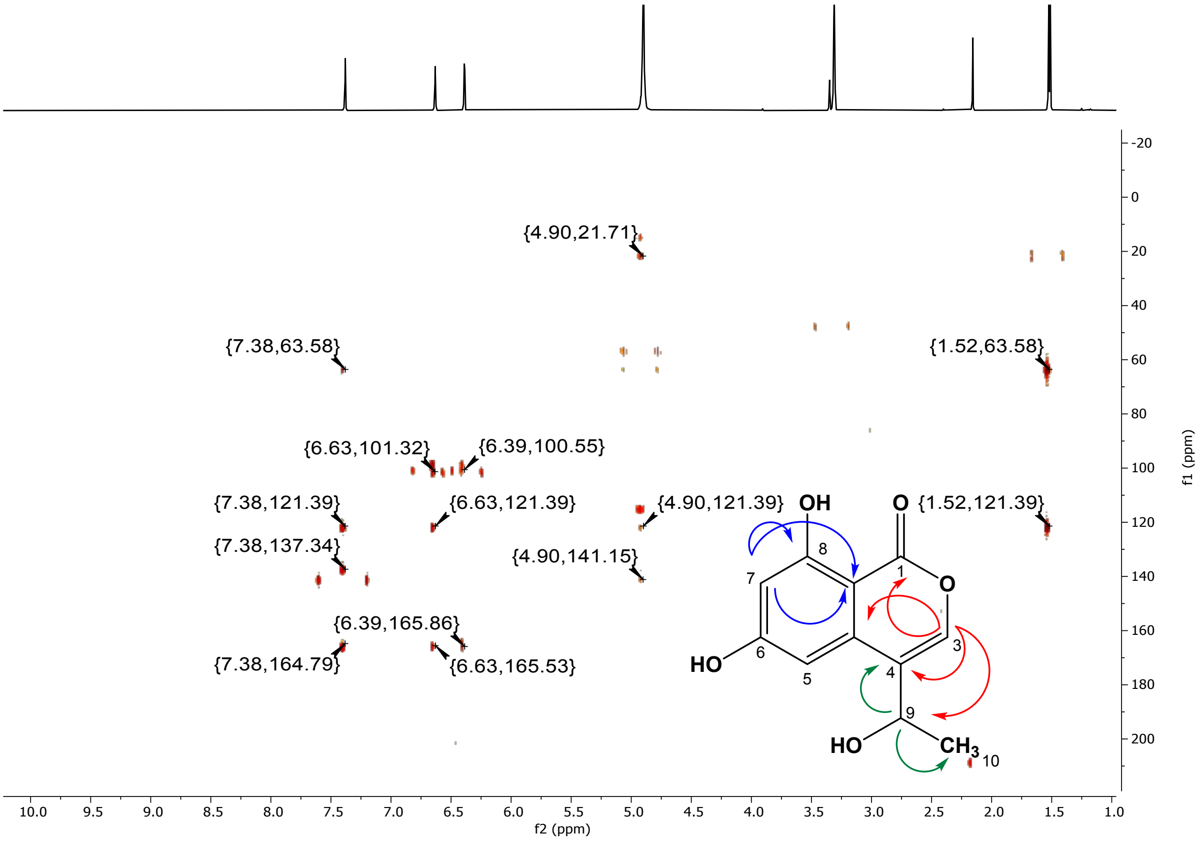

Table S3. ^{1}H and ^{13}C NMR experimental data of compound **3** in comparation to literature data of Sescandelin.

	Sescandelin 500 MHz, $CDCl_3$ (KIMURA; NAKAJIMA; HAMASAKI, 1990)		Compound **3** 500 MHz, $MeOD_4$ (Experimental)	
	**^{13}C δ ppm**	**^{1}H – δ ppm (mult, *J Hz*)**	**^{13}C δ ppm**	**^{1}H – δ ppm (mult, *J Hz*)**
**C-1**	167.2	-	164.7	-
**C-3**	142.9	7.35 (br. s)	141.7	7.38 (br. s)
**C-4**	123.6	-	121.9	-
**C-4a**	139.1	-	137.3	-
**C-5**	102.7	6.66 (d, 2.0)	102.1	6.63 (d, 2.0)
**C-6**	166.6	-	165.3	-
**C-7**	103.2	6.38 (d, 2.0)	101.3	6.39 (d, 2.0)
**C-8**	165.6	-	165.8	-
**C-8a**	100.9	-	100.5	-
**C-9**	65.2	4.90 (br. q, 7.0)	63.5	4.90
**C-10**	24.0	1.45 (d, 7.0)	21.7	1.52 (d, 6.5)
**8-OH**	-	4.60 (s)	-	-
**9-OH**	-	11,39 (s)	-	-

Figure S15. High resolution mass spectrometry of compound **4**

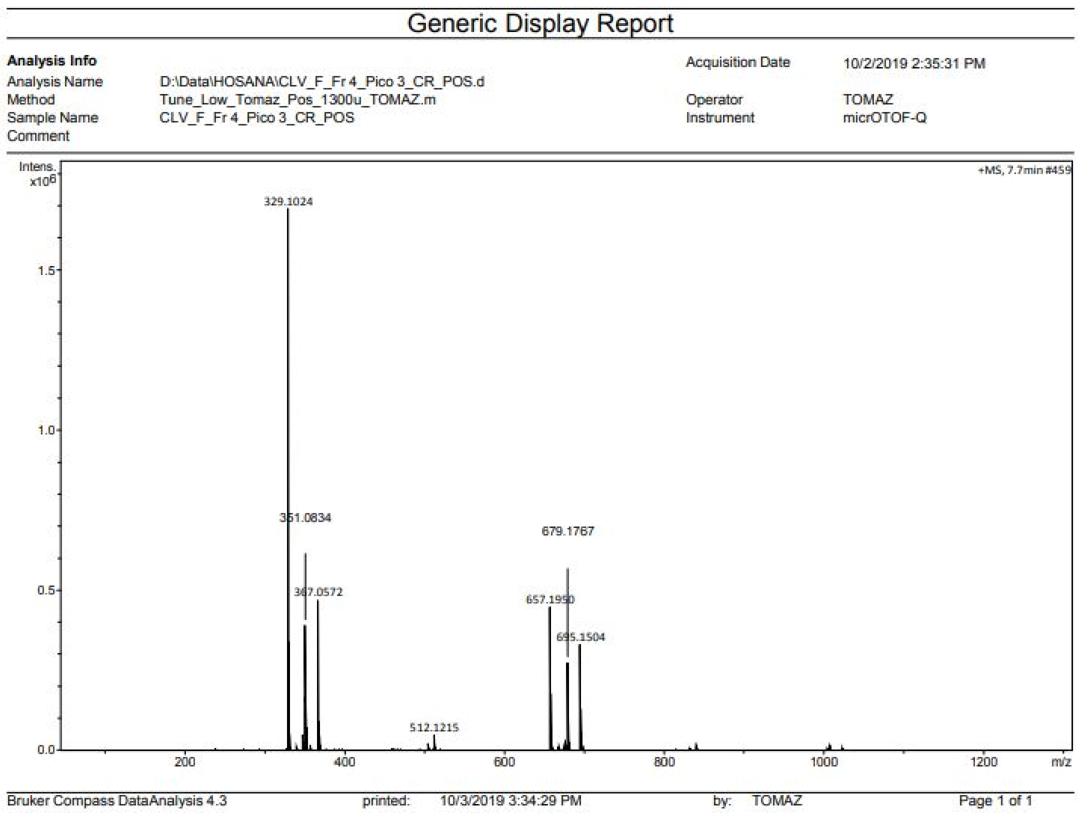

Figure S16. ^{1}H NMR spectrum of compound **4** in $CDCl_3$ measured at 500 MHz.

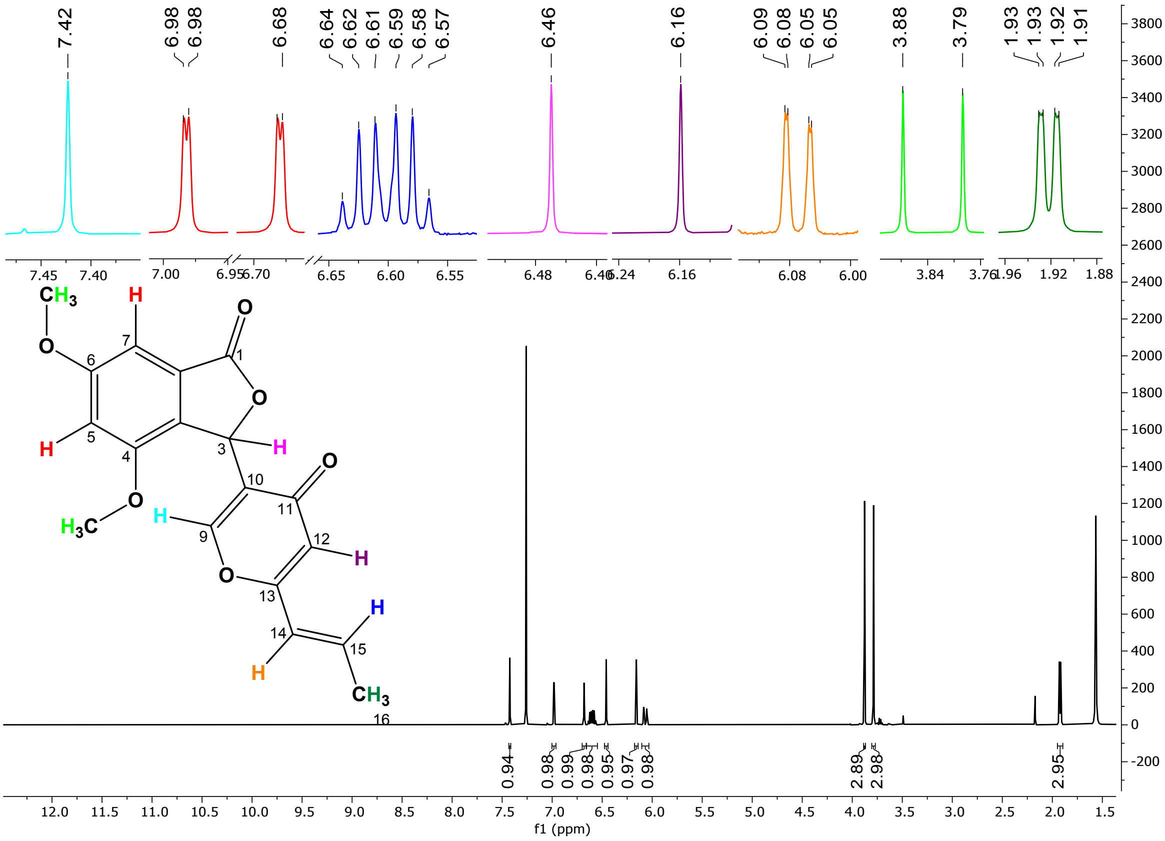

Figure S17. HSQC correlation spectrum of compound **4** in CDCl3 (500 MHz).

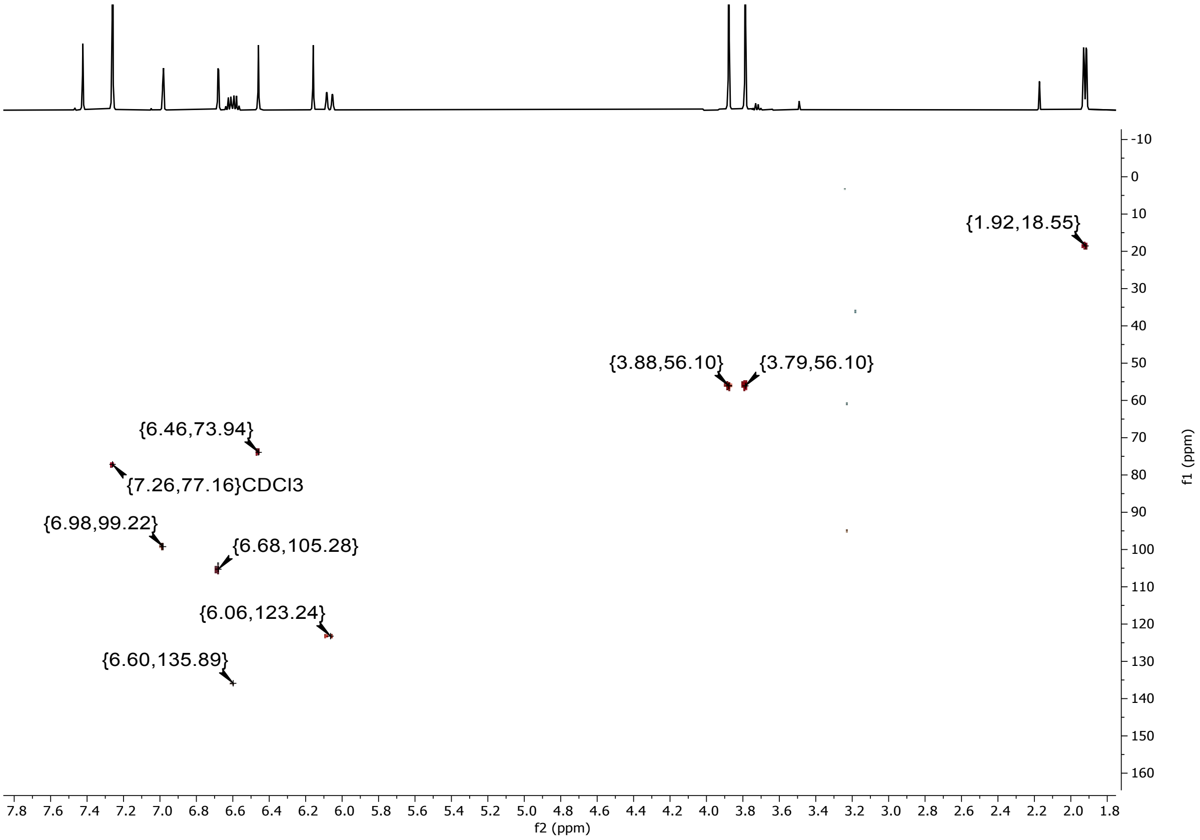

Figure S18. HMBC correlation spectrum of compound **8** in $CDCl_3$ (500 MHz).

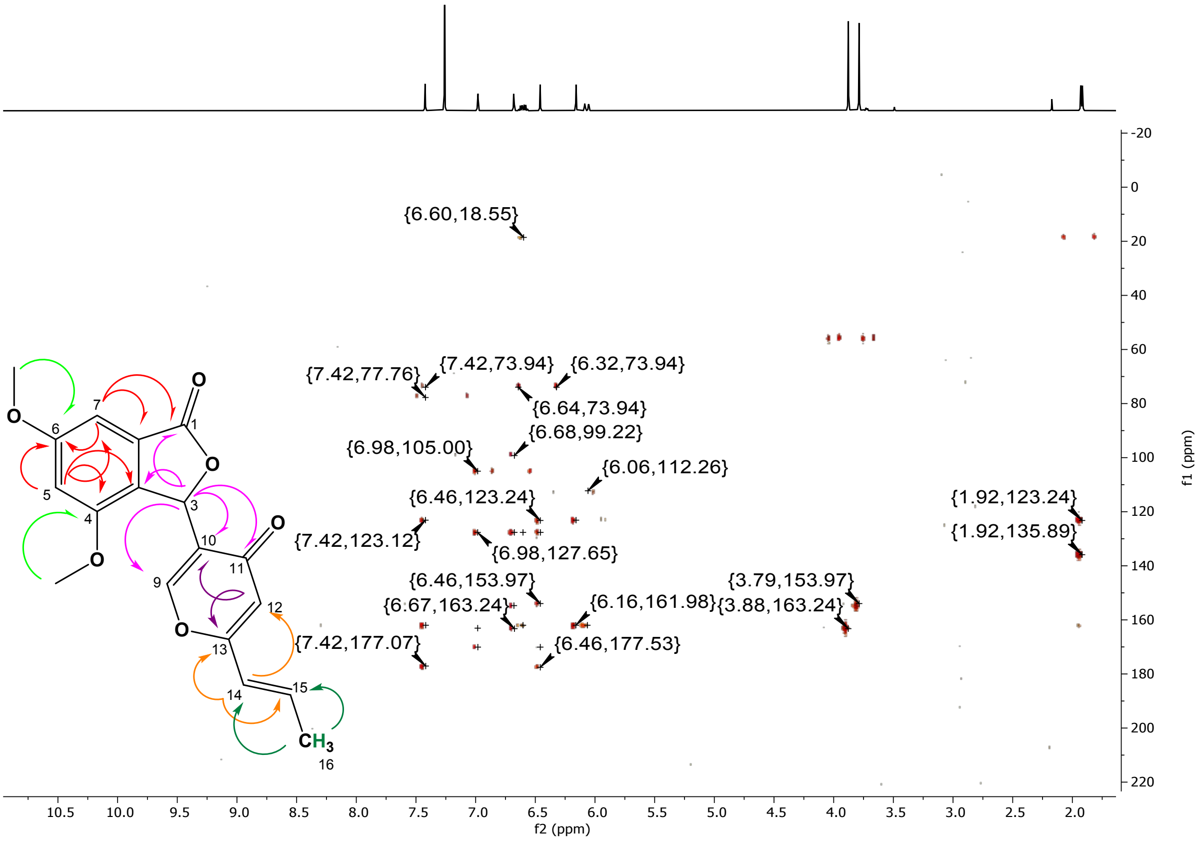

Table S4. ^{1}H and ^{13}C NMR experimental data of compound **4** in comparation to literature data of Vermistatin.

	Vermistatin 400 MHz, $CDCl_3$ (FUSKA et al., 1986)		Compound **4** 500 MHz, CDCl3 (Experimental)	
	**^{13}C δ ppm**	**^{1}H – δ ppm (mult, *J Hz*)**	**^{13}C δ ppm**	**^{1}H – δ ppm (mult, *J Hz*)**
**C-1**	170.0	-	170.5	-
**C-3**	73.6	6.45 (d, 0.6)	73.9	6.46 (s)
**C-3a**	127.7	-	127.6	-
**C-4**	154.9	-	154.7	-
**C-5**	105.1	6.68 (d, 2.0)	105.2	6.68 (d, 2.1)
**C-6**	163.0	-	163.2	-
**C-7**	99.0	6.98 (d, 2.0)	99.2	6.98 (d, 2.1)
**C-7a**	129.3	-	127.6	-
**C-9**	153.9	7.42 (d, 0.6)	153.9	7.42 (s)
**C-10**	123.4	-	123.2	-
**C-11**	177.2	-	177.5	-
**C-12**	112.8	6.15 (dd, 0.5, 0.4)	112.2	6.16 (s)
**C-13**	162.1	-	161.9	-
**C-14**	123.1	6.06 (dq, 15.6, 1.7)	123.1	6.06 (dd, 15.7, 1.9)
**C-15**	135.9	6.60 (dq, 15.6, 6.9, 0.4)	135.8	6.60 (dq, 6.9, 15.7)
**C-16**	18.5	1.92 (ddd, 6.9, 1.7, 0.5)	18.5	1.92 (dd, 6.9, 1.9)
**4-OMe**	55.8	3.79 (s)	56.1	3.79 (s)
**6-OMe**	56.0	3.88 (s)	56.1	3.88 (s)

Method S1 Antibacterial activity against *Cutibacterium acnes*

The minimum inhibitory concentration (MIC, lowest concentration of the compound capable of inhibiting microorganism growth) and the minimum bactericidal concentration (MBC, lowest concentration of the compound at which 99.99% or more of initial inoculum was killed) values were determined in triplicate using the broth microdilution method according to the Clinical Laboratory Standards Institute (CLSI) guidelines [27]. *C. acnes* (ATCC 6919), acquired from American Type Culture Collection, was cultured on Schaedler broth supplemented with vitamin K (1 mg/mL) and hemin (5 mg/mL) for 72h, under anaerobic conditions using anaerobic jar at 37°C. After that, colonies were inoculated in Schaedler agar supplemented with 5% defibrinated sheep blood, vitamin K and hemin for 72h at 37°C under anaerobic conditions. Samples were dissolved in dimethyl sulfoxide (DMSO) followed by dilution in Schaedler medium supplemented with vitamin K and hemin to achieve the concentrations in the range of 100 µg/mL to 0.39 µg/mL for isolated compounds and from 400 to 3.25 µg/mL for crude extracts and fractions. The final DMSO content 5% (v/v) was used as negative control. The inoculum was adjusted to yield cell concentration of $1x10^7$ CFU/mL and penicillin G was used as positive control. The following controls were also included: one inoculated well to allow control of the adequacy of the broth for microorganism growth and one inoculated well free of antimicrobial agent to ensure medium sterility. The microplates were incubated at 37°C for 72h under anaerobic conditions. After incubation, resazurin (aqueous solution 0.02%) was added to the microplates to indicate the microorganism viability. Before that, aliquots were aseptically removed from each well and plated onto Schaeldler agar supplemented with 5% defibrinated sheep blood, vitamin K and hemin and incubated at 37°C under anaerobic conditions for 72h.
